# Toward Industrial Electrosynthesis of Ethylene: Energy‐Efficient and Stable Acetylene Semi‐Hydrogenation on a Copper Phosphide/MXene Electrocatalyst

**DOI:** 10.1002/anie.202518909

**Published:** 2026-01-19

**Authors:** Zeliang Wu, Qihui Guan, Tao Wang, Dongfang Li, Ming Lei, Wei Hong, Shixia Chen, Shijian Wang, Guoxiu Wang, Jun Wang

**Affiliations:** ^1^ School of Chemistry and Chemical Engineering Nanchang University Nanchang 330031 China; ^2^ Centre for Clean Energy Technology School of Mathematical and Physical Sciences Faculty of Science University of Technology Sydney Sydney New South Wales 2007 Australia; ^3^ School of Resources and Environmental Nanchang University Nanchang 330031 China

**Keywords:** Acetylene semi‐hydrogenation, Energy efficiency, Ethylene electrosynthesis, Long‐term stability, Techno‐economic analysis

## Abstract

Electrocatalytic semi‐hydrogenation of acetylene to ethylene (EHAE) using renewable electricity represents a promising alternative approach for ethylene production. However, its relatively low energy efficiency (EE) and insufficient electrocatalyst stability hinder its industrial applications. The conduct a techno‐economic analysis indicates that the EHAE process becomes profitable when the EE exceeds 22.8% at an industrial current density of 0.2 A cm^−2^. Herein, we report a novel electrocatalyst featuring firmly immobilized copper phosphide (Cu_3_P) nanoparticles on MXene nanosheets (Ti_3_C_2_/Cu_3_P) for a stable EHAE process at industrial currents using membrane electrode assembly (MEA) system. Specifically, the Ti_3_C_2_/Cu_3_P electrocatalyst achieves an EE of 23.0% at 0.2 A cm^−2^, demonstrating its potential for practical application and economic viability. The strong interactions between Cu_3_P and Ti_3_C_2_ MXene prevent the agglomeration and dissolution of Cu_3_P nanoparticles during long‐term EHAE process. Notably, in a 4 cm^2^ MEA, Ti_3_C_2_/Cu_3_P catalysts can sustain high performance for 100 h at 1.0 A with an ethylene Faradaic efficiency decay of only 0.051% per hour. Quasi in situ electron paramagnetic resonance spectroscopy and theoretical calculations indicate that Ti_3_C_2_/Cu_3_P facilitates water dissociation and synergistically enhances the adsorption of acetylene and active hydrogen (H^*^), thereby accelerating the kinetics of EHAE process.

## Graphical abstract

Cu_3_P nanoparticles anchored on MXene *via* Ti─O─P bonds exhibit enhanced stability and resist agglomeration during the electrochemical semi‐hydrogenation of C_2_H_2_ to C_2_H_4_. Ti_3_C_2_/Cu_3_P achieves a record energy efficiency of 23.0% at 0.2 A cm^−2^ and stable operation in a 4 cm^2^ MEA for over 100 h at 1.0 A.

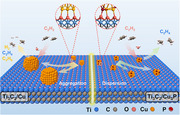

## Introduction

**Figure 1:**
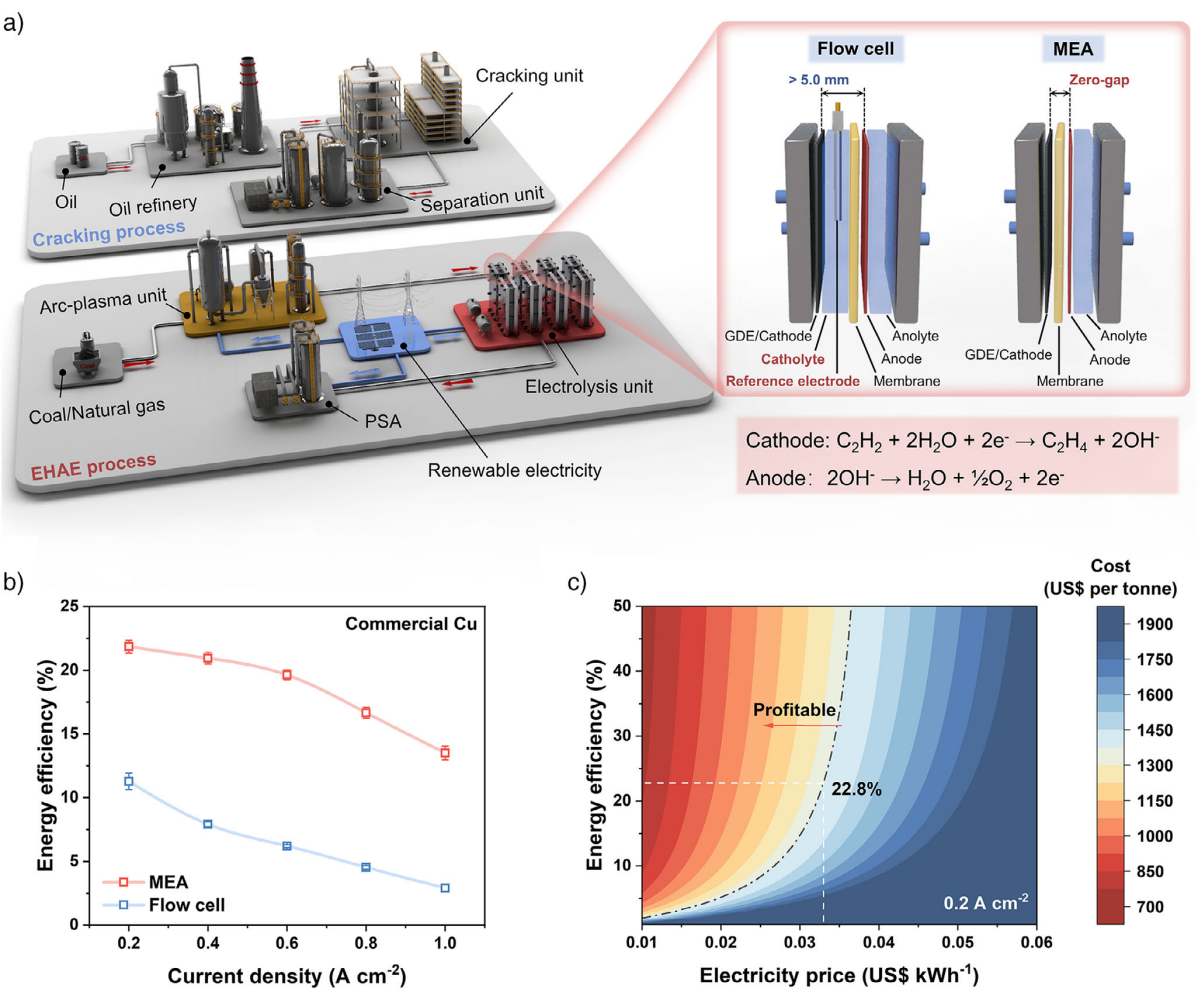
Comparison of electrolyzers in the EHAE process and techno‐economic analysis. (a) Schematic illustration for the EHAE route compared with the traditional cracking route, enlarged comparison of the structures of different electrolyzers in electrolysis unit. (b) Comparison of EE with current density in different electrolyzers for commercial copper nanoparticles. (c) The projected cost of the proposed EHAE process as a function of energy efficiency and electricity cost at 0.2 A cm^−2^. The area above the dashed‐dotted grey line indicates a profitable region.

Ethylene (C_2_H_4_) is the most important bulk commodity in the modern chemical industry, primarily produced through the steam cracking of oil and shale gas.^[^ ^1^, ^2^ ^]^ Given the global transition toward carbon neutrality, the high energy consumption and massive carbon emissions associated with these processes necessitate the exploration of alternatives to zero‐carbon emission technology for C_2_H_4_ production.^[^ ^3^ ^]^ The electrocatalytic semi‐hydrogenation of acetylene (C_2_H_2_) to C_2_H_4_ (EHAE), utilizing water as the proton source and powered by renewable electricity, has emerged as a promising petroleum‐independent route due to its economic viability and environmental friendliness (Figure 1a).^[^ ^4^, ^5^, ^6^, ^7^ ^]^ For industrial implementation, it is essential to develop electrocatalysts and reactor systems with significantly enhanced energy efficiency (EE), activity, and selectivity toward C_2_H_4_.

To date, most EHAE studies are conducted in three‐electrode flow cell systems using gas‐diffusion electrodes (GDEs).^[^ ^1^, ^8^, ^9^, ^10^ ^]^ Satisfactory C_2_H_4_ Faradaic efficiency (FE) values (>90%) are readily achieved at relatively large current densities (>0.2 A cm^−2^) by optimizing copper‐based electrocatalysts.^[^ ^11^, ^12^, ^13^ ^]^ In our previous work, we achieved a C_2_H_4_ FE exceeding 90% at current densities above 0.5 A cm^−2^ by introducing oxygen/nitrogen vacancies into nano‐copper electrocatalysts.^[^ ^14^, ^15^ ^]^ However, the electrolyte in the gap between the cathode and anode in flow cells causes elevated resistance at large current densities, which require higher overpotentials and result in low full‐cell EE values. Notably, the FE value and cell voltage, a key trade‐off pair, determine the commercial viability of the EHAE process.^[^ ^16^ ^]^ In contrast, zero‐gap membrane electrode assemblies (MEAs) featuring near zero‐gap between electrodes can significantly reduce the internal resistance and enable higher current density and lower cell voltage (Figure 1a). Using commercial copper nanoparticles as the control electrocatalyst, we compare the variation of EE with current density within two reaction systems. The EE value in the MEA system reached 21.8% at 0.2 A cm^−2^, approximately 1.9 times higher than that of the flow cell system, and the difference becomes even more pronounced at higher current density (4.6 times at 1.0 A cm^−2^) (Figure 1b). Furthermore, the techno‐economic analysis (TEA) demonstrates that the EHAE process is profitable when the EE exceeding 22.8% at a current density of 0.2 A cm^−2^ with electricity price of US$ 0.033 per kWh (Figure 1c and Supplementary Note 1). Additionally, the profitability range further expand as the EE increases and the price of renewable electricity decreases.

In previous EHAE studies, the electrocatalyst stability is commonly overlooked compared to the reaction activity and selectivity.^[^ ^10^, ^17^, ^18^ ^]^ Most reported long‐term stability was below 55 h at industry‐scale current densities (0.2 A cm^−2^), which remains insufficient for potential commercialization.^[^ ^1^, ^8^, ^12^ ^]^ Generally, anchoring catalysts on appropriate matrices can stabilize and disperse the active species *via* strong catalyst‐support interactions, thereby improving both catalytic activity and stability.^[^ ^19^, ^20^, ^21^ ^]^ 2D transition metal carbides, known as MXenes, are considered ideal matrix owing to their large surface area, superior conductivity, and tunable surface chemistry.^[^ ^22^, ^23^, ^24^, ^25^ ^]^ The surface terminal groups of MXenes are negatively‐charged, which reinforce electrostatic interactions with positively‐charged metal cations, therefore promoting high dispersion and binding of metal nanoparticle catalysts on MXene.^[^ ^21^, ^25^, ^26^ ^]^ Zang et.al. demonstrated that loading metal nanoclusters onto MXene effectively suppresses their aggregation during the electroreduction process, thereby improving the stability of the electrocatalyst.^[^ ^27^ ^]^ Nevertheless, the weak interactions mediated solely by the surface functional groups of MXenes are unlikely to ensure structural robustness under industrially relevant current densities. A more resilient interface can be obtained by introducing metal compounds capable of forming covalent bonds with the support.^[^ ^28^, ^29^ ^]^ Copper phosphides represent a rational candidate due to their demonstrated structural stability and favorable electrochemical performance under hydrogenation conditions.^[^ ^30^ ^]^ Therefore, anchoring copper phosphides onto MXene to construct a covalently bonded interface is expected to offer significant promise for enabling an efficient and stable EHAE process—yet this strategy remains unexplored.

Herein, we present a stable electrocatalyst featuring firmly anchored copper phosphide (Cu_3_P) nanoparticles on MXene support (Ti_3_C_2_/Cu_3_P) for efficient C_2_H_4_ electrosynthesis through EHAE process. The strong interactions between Cu_3_P and Ti_3_C_2_ MXene *via* covalent Ti─O─P bonds facilitate the uniform dispersion of Cu_3_P and prevent its aggregation and dissolution during long‐term operation. Moreover, Quasi in situ electron paramagnetic resonance (EPR) spectroscopy and theoretical calculations reveal that Ti_3_C_2_/Cu_3_P facilitates water dissociation to produce active H^*^, and the enhanced adsorption of C_2_H_2_ and H^*^ accelerates semi‐hydrogenation kinetics. As a result, the Ti_3_C_2_/Cu_3_P electrocatalyst delivers a high EE value of 23.0% at a current density of 0.2 A cm^−2^ with a C_2_H_4_ FE of 94.3%. By inputting these operation parameters measured at different current densities (0.2‐1.0 A cm^−2^), we predict that the EHAE process is economically feasible. Furthermore, in a 4 cm^2^ MEA system, Ti_3_C_2_/Cu_3_P exhibits remarkable stability exceeding 100 h at 1.0 A with a C_2_H_4_ FE decay of only 0.051% per hour. This work provides insights into the industrial implementation and economic feasibility of electrosynthesis of C_2_H_4_ from C_2_H_2_.

## Results and Discussions

### Synthesis and Characterizations of Catalysts

**Figure 2:**
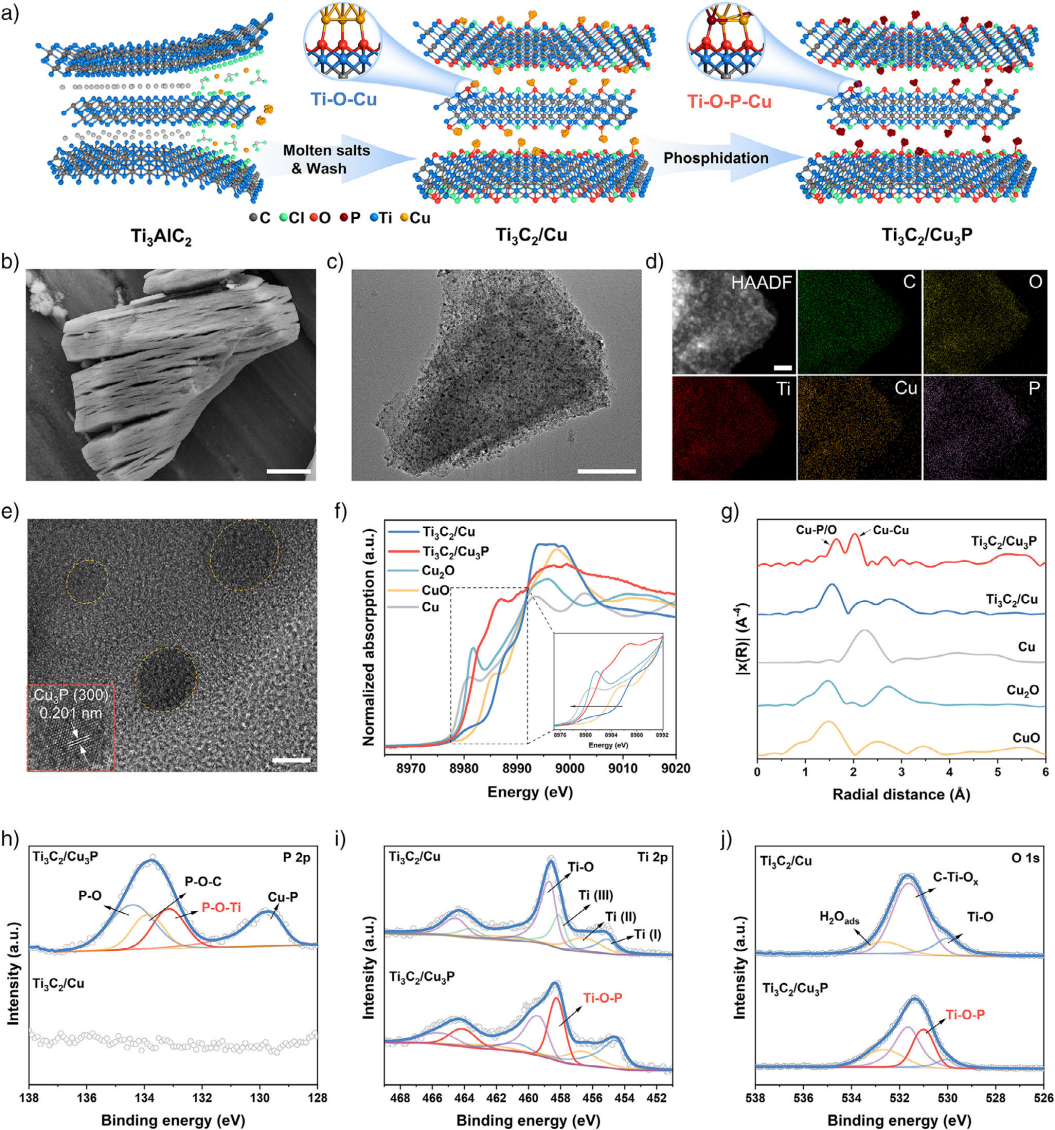
The synthesis and structural characterization of Ti_3_C_2_/Cu_3_P. (a) Schematic of the synthesis process of Ti_3_C_2_/Cu_3_P. (b) SEM image (scale bars: 2 µm), (c) TEM image (scale bars: 200 nm), (d) HAADF‐STEM image and corresponding elemental mappings (scale bars: 50 nm), and (e) HRTEM image (scale bars: 5 nm) of Ti_3_C_2_/Cu_3_P. Normalized Cu K‐edge XANES (f) and Fourier transform of EXAFS (g) spectra of Ti_3_C_2_/Cu, Ti_3_C_2_/Cu_3_P, and references. High‐resolution XPS spectra of P 2p (h), Ti 2p (i), and O 1s (j).

The synthesis procedures for Ti_3_C_2_/Cu_3_P involve etching the Ti_3_AlC_2_ phase through redox reactions between Al^3+^ and CuCl_2_ as a Lewis acid molten salt (Figure 2a).^[^ ^23^ ^]^ Subsequently, Cu_3_P nanoparticles were anchored onto Ti_3_C_2_ MXene through a facile phosphidation process. Each step in the preparation process of Ti_3_C_2_/Cu_3_P was confirmed by powder X‐ray diffraction (PXRD) patterns (Figure S1 and Supplementary Note 2). The morphology at each stage was also characterized using scanning electron microscopy (SEM) (Figures S2 and S3). EDS analysis further confirms that Al is effectively exchanged by Cu (Figure S4 and Supplementary Note 3). After complete etching, the initial dense structure evolved into a typical accordion‐like MXene structure, which remained stable throughout the phosphidation process (Figure 2b).^[^ ^23^, ^31^ ^]^ Transmission electron microscopy (TEM) images revealed the lamellar microstructures of both Ti_3_C_2_/Cu and Ti_3_C_2_/Cu_3_P, which can be attributed to the fact that the generated AlCl_3_ vapors facilitated the delamination of MXene (Figures S5 and S6).^[^ ^23^ ^]^ Furthermore, high‐resolution TEM (HRTEM) images showed the (002) facet of Ti_3_C_2_ with an interlayer spacing of 1.2 nm and the (111) facet of Cu with an interlayer spacing of 2.08 Å (Figure S7).^[^ ^32^ ^]^ Upon ultrasonic exfoliation, Cu_3_P nanoparticles were evenly dispersed on the surfaces of ultrathin MXene nanosheets (Figure 2c). Elemental mapping analysis confirmed the uniform distributions of C, O, Ti, Cu, and P elements throughout Ti_3_C_2_/Cu_3_P (Figure 2d). The lattice fringes with an interlayer spacing of 2.01 Å belonged to the (300) facet of Cu_3_P (Figure 2e).^[^ ^33^, ^34^ ^]^ The specific surface area slightly decreased following phosphidation (Figure S8). The inductively coupled plasma‐mass spectrometry (ICP‐MS) results demonstrated the Cu content of about 17.2 wt% on Ti_3_C_2_/Cu_3_P.

In the Cu K‐edge X‐ray absorption near edge structure (XANES) spectra, the absorption edge of Ti_3_C_2_/Cu resembled that of the CuO reference, indicating a high oxidation state of Cu.^[^ ^35^ ^]^ After phosphidation, the absorption edge of Ti_3_C_2_/Cu_3_P in the Cu K‐edge XANES spectra shifted to lower energy and approached that of the Cu_2_O reference, suggesting a partial reduction of Cu^2+^ to Cu^+^ (Figure 2f).^[^ ^36^ ^]^ Notably, a peak near 8986 eV was preserved, similar to CuO reference, which indicates the coexistence of Cu─P and Cu─O coordinations in Ti_3_C_2_/Cu_3_P. The Fourier transform from extended X‐ray absorption fine structure (EXAFS) spectra at the Cu K‐edge of Ti_3_C_2_/Cu showed two peaks at 1.56 and 2.23 Å, corresponding to Cu─O and Cu─Cu bonds, respectively (Figure 2g).^[^ ^35^ ^]^ Furthermore, the spectra of Ti_3_C_2_/Cu_3_P exhibited two peaks at 1.65 and 2.02 Å, which were assigned to the Cu─P/O and Cu─Cu bonds, respectively.^[^ ^37^ ^]^

X‐ray photoelectron spectroscopy (XPS) analysis elucidated the surface composition and chemical states of Ti_3_C_2_/Cu and Ti_3_C_2_/Cu_3_P. The Cu 2p peaks at 935.3/955.1 eV were assigned to the Cu─O bond, formed by the reaction of Cu^2+^ and oxygen‐containing surface groups (Figure S9 and Supplementary Note 4). Compared to Ti_3_C_2_/Cu, the peak intensity of Ti_3_C_2_/Cu_3_P at 932.5/952.3 eV markedly increased, indicating the formation of Cu─P bonds during the phosphidation process.^[^ ^34^ ^]^ The P 2p spectrum displayed Cu─P and P─O─Ti bonds at 129.7 and 133.1 eV, respectively (Figure 2h).^[^ ^38^ ^]^ For Ti_3_C_2_/Cu, the Ti 2p signal exhibited multiple Ti valence states and Ti─O bonds (Figure 2i).^[^ ^35^ ^]^ After phosphidation, the characteristic peaks for Ti (III) disappeared, and a pair of peaks emerged at 458.3/464.2 eV, corresponding to the Ti─O─P bond.^[^ ^38^, ^39^ ^]^ Furthermore, the Ti─O─P bond was also confirmed by the O 1s signal at 531.0 eV in Ti_3_C_2_/Cu_3_P (Figure 2j).^[^ ^38^ ^]^ These results indicated that the Cu_3_P nanoparticles were bonded to the MXene support through Ti─O─P and Cu─O bonds.^[^ ^38^, ^39^ ^]^

### Electrocatalytic Semi‐Hydrogenation of C_2_H_2_ to C_2_H_4_

**Figure 3:**
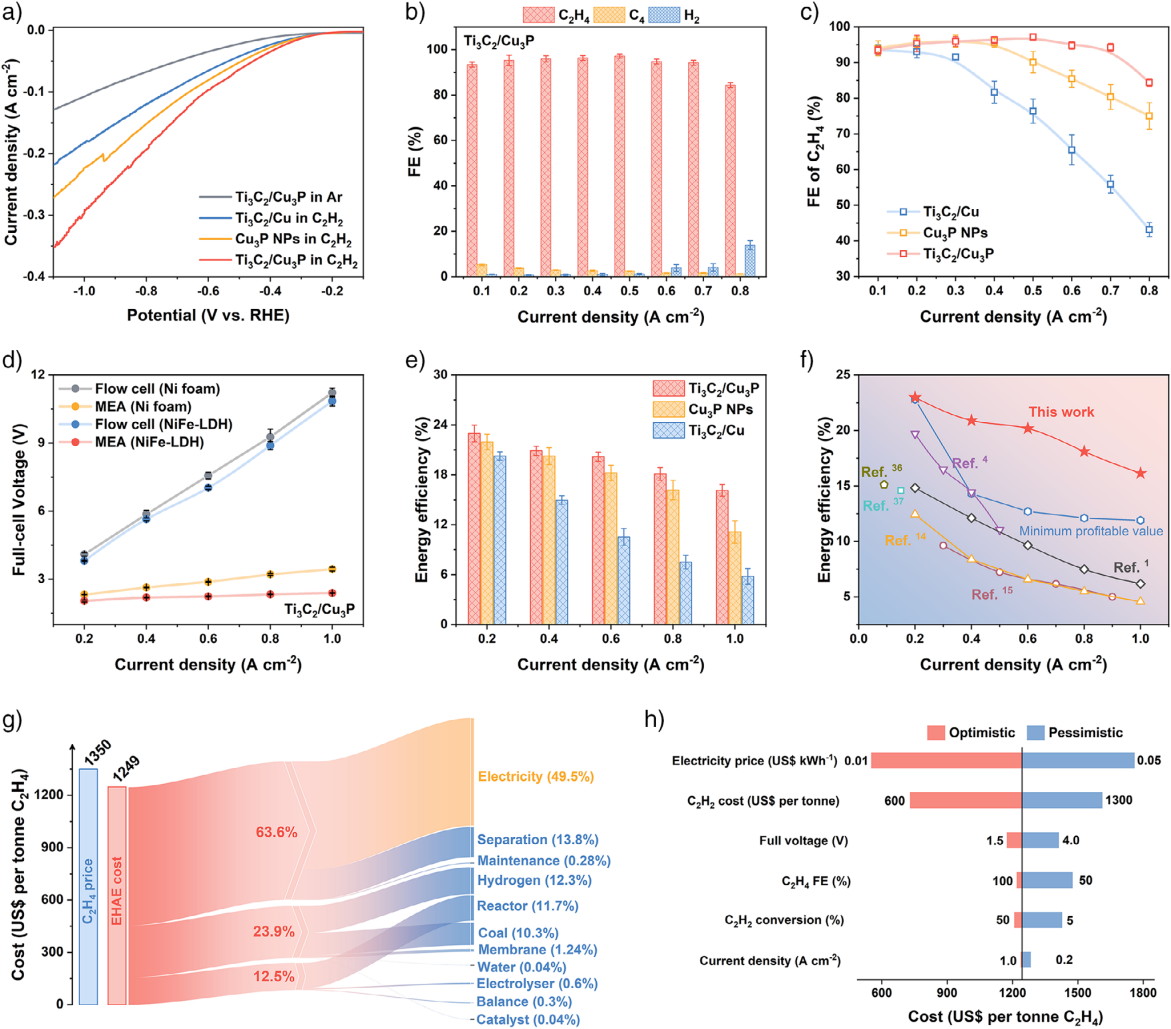
Electrocatalytic performance and cost analysis of the process. In a three‐electrode flow cell. (a) LSV curves for Ti_3_C_2_/Cu_3_P, Cu_3_P NPs, and Ti_3_C_2_/Cu. (b) The FE of EHAE products at different current densities of Ti_3_C_2_/Cu_3_P. (c) The FE of C_2_H_4_ at different current densities for Ti_3_C_2_/Cu_3_P, Cu_3_P NPs, and Ti_3_C_2_/Cu. (d) The full‐cell voltage of Ti_3_C_2_/Cu_3_P in the different electrolyzers and anode. (e) The energy efficiency of Ti_3_C_2_/Cu_3_P and Ti_3_C_2_/Cu at different current densities in MEA. (f) A comparison of the reported full‐cell energy efficiency. (g) The subdivided cost of the EHAE process under a current density of 0.6 A cm^−2^, full‐cell voltage of 2.24 V, and C_2_H_4_ FE of 90.9% at the given electricity price of US$ 0.033/kWh. (h) Single‐variable sensitivity analysis for the production cost of C_2_H_4_.

The electrocatalytic performance of EHAE was first evaluated in a standard three‐electrode flow cell. Linear sweep voltammetry (LSV) curves demonstrated that the addition of C_2_H_2_ led to a higher responsive current density than that of Ar (Figure 3a).^[^ ^17^ ^]^ Moreover, Ti_3_C_2_/Cu_3_P delivered a cathodic current density of 350.4 mA cm^−2^ at −1.1 V (vs. the reversible hydrogen electrode, RHE), surpassing the value of 271.3 mA cm^−2^ for Cu_3_P nanoparticles (Cu_3_P NPs) and 218.0 mA cm^−2^ for Ti_3_C_2_/Cu. These results showed the superior activity of Ti_3_C_2_/Cu_3_P to drive C_2_H_2_ electrochemical hydrogenation. The superior EHAE activity of Ti_3_C_2_/Cu_3_P remained even after normalization by the electrochemical surface area (ECSA) (Figures S10 and S11). Moreover, steady‐state chronoamperometric measurements of C_2_H_2_ semi‐hydrogenation were conducted at various cathodic current densities ranging from 0.1 to 0.8 A cm^−2^. Notably, the Ti_3_C_2_ matrix without Cu nanoparticles predominantly produced hydrogen (H_2_), suggesting that Cu served as the primary active site for the EHAE process (Figure S12). In contrast, C_2_H_4_ was the main hydrogenation product on Ti_3_C_2_/Cu_3_P, along with minor amounts of C_4_ (mainly 1,3‐butadiene) and H_2_ (Figure 3b). Across a wide range of current density from 0.2 to 0.7 A cm^−2^, the FE for C_2_H_4_ consistently exceeded 94.2%, outperforming both Ti_3_C_2_/Cu and Cu_3_P NPs (Figure 3c and Figure S13). Additionally, the C_2_H_4_ yield on the Ti_3_C_2_/Cu_3_P catalyst reached 12.59 mmol h^−1^ cm^−2^ at a current density of 0.8 A cm^−2^, surpassing that of Ti_3_C_2_/Cu (6.44 mmol h^−1^ cm^−2^) and Cu_3_P NPs (11.20 mmol h^−1^ cm^−2^), as well as most previously reported electrocatalysts (Figures S14 and S15 and Table S1).^[^ ^1^, ^4^, ^13^, ^40^ ^]^ Meanwhile, the Ti_3_C_2_/Cu_3_P catalyst demonstrated an outstanding capability to inhibit HER and over‐hydrogenation (Figures S16 and S17 and Supplementary Notes 5–6).

We further investigated the electrocatalytic performance of EHAE in a two‐electrode zero‐gap MEA system (Figure S18). As a result, the C_2_H_4_ FE reached 94.3% at 0.2 A cm^−2^ and remained as high as 90.9% at 0.6 A cm^−2^, which is comparable to that in the flow cell (Figure S19 and Table S2). Notably, although the use of a more efficient oxygen evolution reaction (OER) catalyst can partially decrease the full‐cell voltage, the intrinsic structural advantages of the electrolyzer play a more decisive role in determining its operational performance. At a current density of 0.2 A cm^−2^ without iR compensation, the full‐cell voltage was significantly reduced from 3.81 V to 2.02 V, and this drop became more significant at higher current densities (Figure 3d). The reduction in full‐cell voltage substantially enhanced the EE for C_2_H_4_ production, increasing from 12.4% to 23.0%, which is beneficial for reducing the overall energy consumption of the EHAE process (Figure S20). Additionally, the Ti_3_C_2_/Cu_3_P catalyst in the MEA system consistently surpassed Cu_3_P NPs and Ti_3_C_2_/Cu in both electrochemical activity and C_2_H_4_ selectivity, which is consistent with the results in the flow cell (Figures S21–S23 and Supplementary Notes 7–8). Notably, even at an ultrahigh current density of 1.0 A cm^−2^, Ti_3_C_2_/Cu_3_P maintained an EE value of 16.1%, outperforming Cu_3_P NPs (11.1%), Ti_3_C_2_/Cu (5.8%), and other reported electrocatalysts (Figure 3e,f and Table S3).^[^ ^1^, ^4^, ^14^, ^15^, ^40^, ^41^ ^]^ Note that the calculation method for the EE value has been standardized based on reported data. Additionally, the C_2_H_4_ EE of Ti_3_C_2_/Cu_3_P at different current densities consistently exceeded the minimum threshold required for profitability, thereby highlighting the considerable economic potential of Ti_3_C_2_/Cu_3_P in the EHAE process (Figure S24 and Supplementary Note 9).

The detailed cost analysis of the EHAE process has been performed (Tables S4 and S5). As shown in Figure 3g, electricity cost represents the largest component, accounting for 49.5%. Thanks to the ongoing decrease in renewable electricity prices and improvements in system energy efficiency, the overall C_2_H_4_ production cost for the EHAE process is expected to be further reduced. Moreover, the single‐variable sensitivity analysis also revealed that the production cost was most sensitive to fluctuations in the electricity price (Figure 3h). Moreover, the price of C_2_H_2_ feedstock is also a critical factor, which can potentially be reduced by advancements in arc electric arc pyrolysis technology for coal and natural gas in the near future. From the perspective of electrochemical process, the design of efficient electrocatalysts should not only aim to achieve high C_2_H_4_ FEs at high current densities, but also focus on reducing the full‐cell voltage and improving long‐term stability.

**Figure 4:**
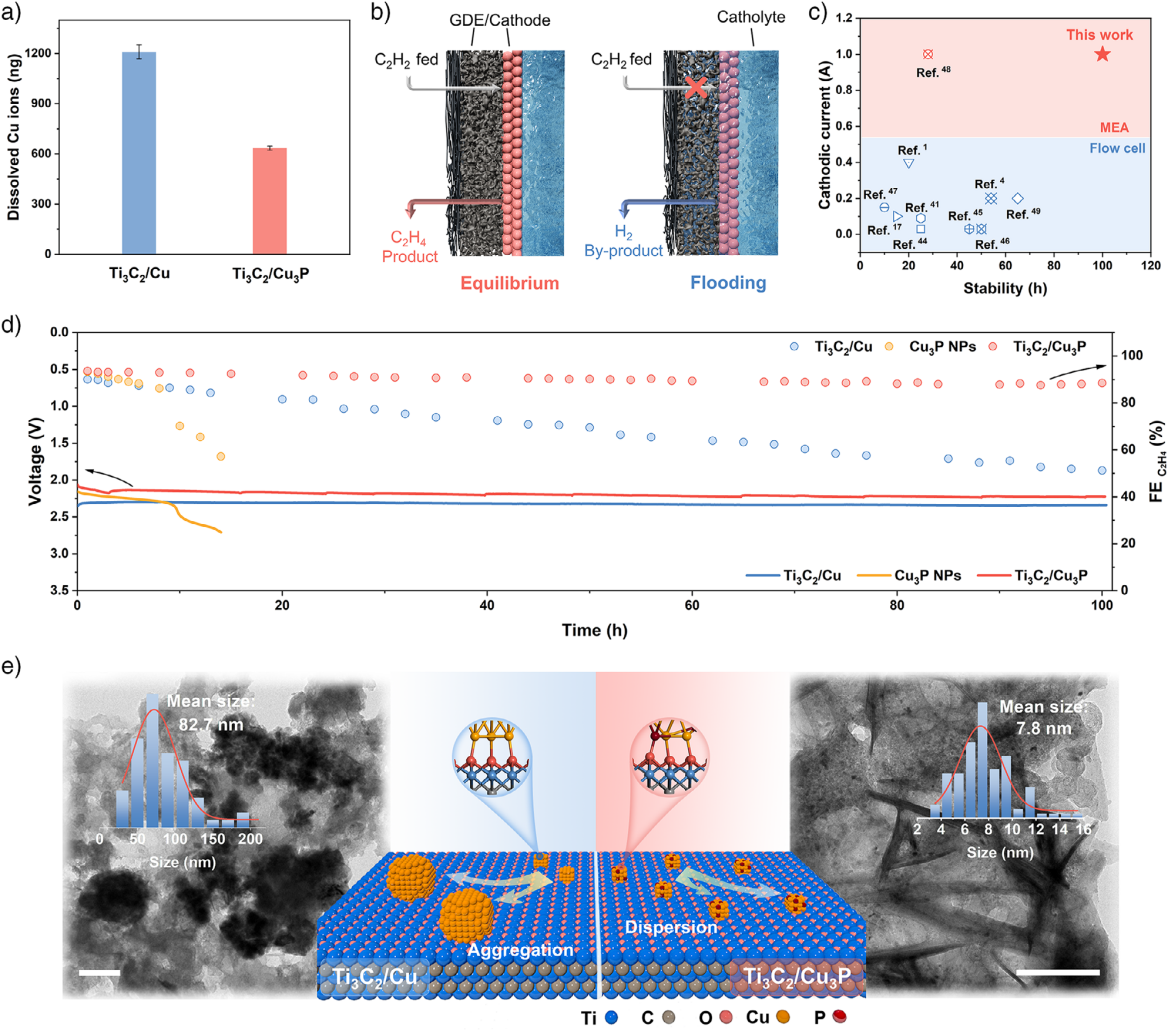
Investigation of catalyst stability. (a) the dissolution of Cu in Ti_3_C_2_/Cu and Ti_3_C_2_/Cu_3_P electrode at 1.0 A for 10 h. (b) Schematic illustration of flow cell at a three‐phase interface. (c) Comparison of the C_2_H_2_ semi‐hydrogenation stability over Ti_3_C_2_/Cu_3_P with previously reported works. (d) Stability test of the 4 cm^2^ MEA measured at 1 A, with a flow rate of 20 mL min^−1^. (e) TEM images, particle size distribution, and schematic illustration of Ti_3_C_2_/Cu and Ti_3_C_2_/Cu_3_P after long‐term stability tests, scale bars: 200 nm.

To further elaborate the foundation for practical applications, stability tests were conducted in flow cell and MEA with a larger electrode area (4 cm^2^). Under a current of 1.0 A for 10 h in the flow cell, ICP‐MS analysis of the catholyte confirmed the dissolution of Cu from catalyst. Notably, the Cu dissolution from Ti_3_C_2_/Cu_3_P was markedly lower than that from Ti_3_C_2_/Cu, indicating that Ti_3_C_2_/Cu_3_P exhibits superior anti‐dissolution performance under industrial‐level current densities (Figure 4a and Figure S25). Furthermore, severe flooding of the cathodic GDE was consistently observed during the stability tests (Figure S26). In flow cells, the applied electrocatalysts are in direct contact with the electrolyte, rendering a delicate balance at the tri‐interface that is highly susceptible to pressure fluctuations on either side.^[^ ^42^ ^]^ When the interface is at equilibrium, C_2_H_2_ can permeate through the gas diffusion layer (GDL) and rapidly access the reaction interface. However, over time, flooding becomes inevitable, causing electrolyte intrusion into the electrocatalyst and impeding C_2_H_2_ diffusion (Figure 4b).^[^ ^43^ ^]^ In contrast, the cathode in MEA requires a minimal volume of electrolyte, and water molecules are supplied through the anion exchange membrane (AEM), thus minimizing the risk of flooding. Therefore, we conducted further evaluation using a 4 cm^2^ MEA system. Under a pure C_2_H_2_ flow, the full cell current reached 1.73 A at a voltage of 2.14 V (Figure S27). Furthermore, the Ti_3_C_2_/Cu_3_P catalyst demonstrated exceptional stability over 100 h at 1.0 A, consistently maintaining high C_2_H_4_ FEs above 88.5% and outperforming most reported electrocatalysts (Figure 4c).^[^ ^1^, ^4^, ^17^, ^41^, ^44^, ^45^, ^46^, ^47^, ^48^, ^49^ ^]^ In contrast, Cu_3_P NPs and Ti_3_C_2_/Cu rapidly deactivated within the first 10 h. Specifically, Ti_3_C_2_/Cu showed an average C_2_H_4_ FE decay rate of 0.39% per hour, which is significantly higher than that of Ti_3_C_2_/Cu_3_P (0.051% per hour) (Figure 4d). In addition, during the stability test, Ti_3_C_2_/Cu_3_P exhibited a C_2_H_2_ conversion decay rate of 0.048% per hour while maintaining C_2_H_4_ selectivity consistently above 93.5% (Figure S28). Notably, after 100 h of continuous operation, the MEA cell was disassembled, and no signs of flooding were detected behind the cathode (Figure S29).

To further elucidate the cause of electrocatalyst deactivation, TEM characterizations were recorded for both Ti_3_C_2_/Cu and Ti_3_C_2_/Cu_3_P after the long‐term stability test. The average size of Cu nanoparticles in Ti_3_C_2_/Cu significantly increased from 4.6 nm to 82.7 nm (Figure 4e left). In sharp contrast, for Ti_3_C_2_/Cu_3_P, the average size of Cu_3_P nanoparticles only slightly increased from 6.6 nm to 7.8 nm (Figure 4e right and Figures S30 and S31). Therefore, compared with the pronounced aggregation observed in Ti_3_C_2_/Cu, the aggregation propensity on Ti_3_C_2_/Cu_3_P has been effectively suppressed, thereby preventing rapid deactivation and enhancing long‐term stability.

### Mechanism of the EHAE Conversion

**Figure 5:**
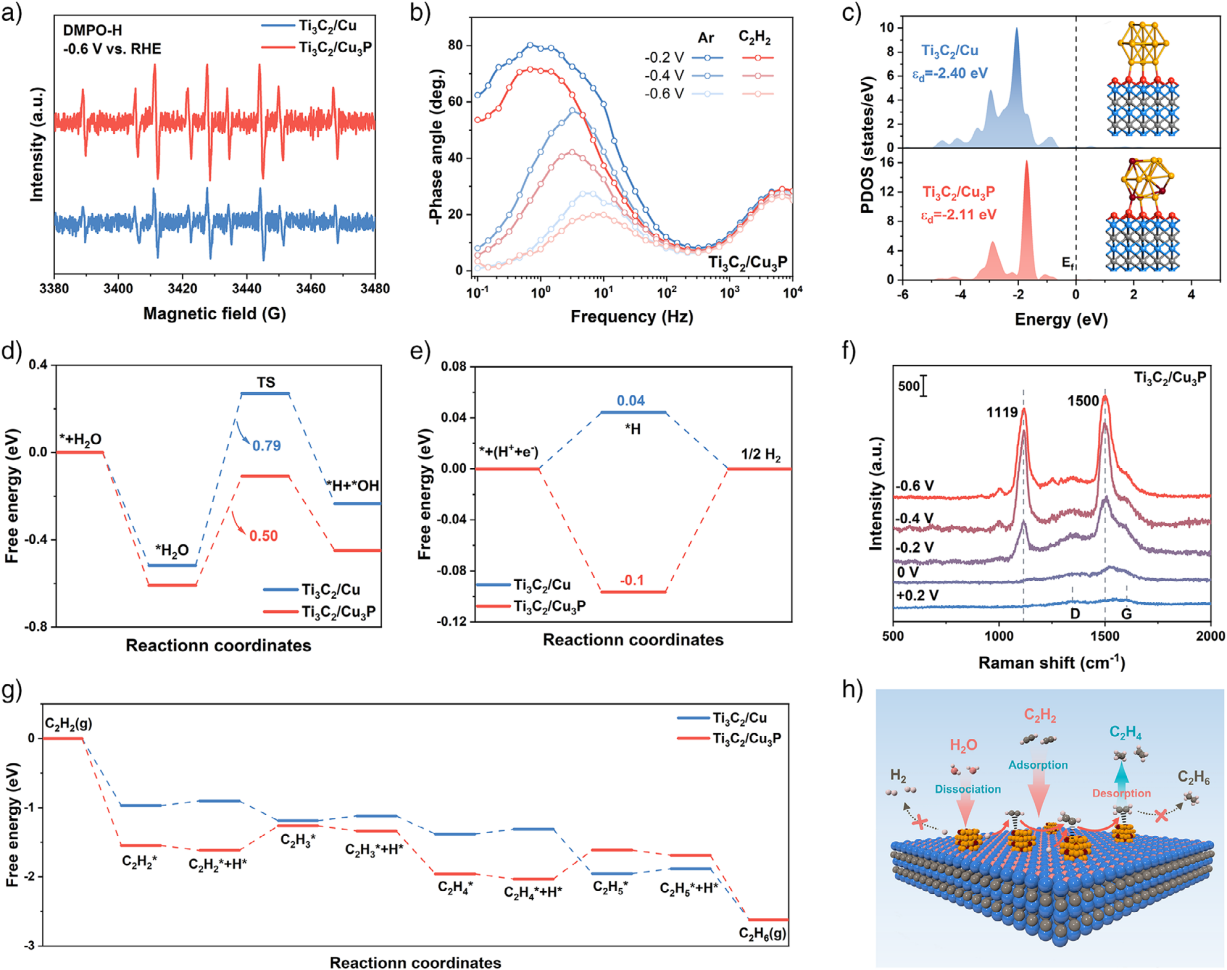
Mechanism of C_2_H_4_ production. (a) Quasi in situ EPR measurements of Ti_3_C_2_/Cu and Ti_3_C_2_/Cu_3_P at −0.6 V vs. RHE. (b) Bode phase plots with Ar and C_2_H_2_ at various potentials for Ti_3_C_2_/Cu_3_P. **(c**) The projected density of states (PDOS) and corresponding structure models for Ti_3_C_2_/Cu and Ti_3_C_2_/Cu_3_P. (d, e) Free energy diagram for (d) H_2_O dissociation and (e) HER pathways. **(f**) In situ Raman spectra at different potentials on Ti_3_C_2_/Cu_3_P electrodes. (g) Free energy profiles for the hydrogenation of C_2_H_2_ on Ti_3_C_2_/Cu and Ti_3_C_2_/Cu_3_P. (h) Schematic illustration of the EHAE pathway on Ti_3_C_2_/Cu_3_P.

To elucidate the underlying mechanism for the superior performance of the EHAE conversion on Ti_3_C_2_/Cu_3_P, we conducted a series of experimental characterizations and theoretical calculations. First, the H^*^ source in the EHAE process was identified. The kinetic isotope effect (KIE) values (H/D) for Ti_3_C_2_/Cu_3_P consistently exceeded 2, indicating that water dissociation is kinetically involved as the rate‐determining step in the EHAE (Figure S32). Moreover, increasing the electrolyte pH resulted in a corresponding rise in C_2_H_4_ FE and suppression of HER, indicating that C_2_H_2_ preferentially undergoes hydrogenation *via* H^*^ dissociated from water, rather than through the conventional proton‐coupled electron transfer (PCET) processes at high pH values (Figure S33).^[^ ^5^, ^12^ ^]^ Furthermore, the hydrogen radicals (denoted as •H) were detected by Quasi in situ electron paramagnetic resonance (EPR) measurements using 5,5‐dimethyl‐1‐pyrroline‐*N*‐oxide (DMPO) as the spin‐trapping agent (Figure 5a and Figure S34). Under Ar flow, the stronger DMPO‐H signal intensity observed for Ti_3_C_2_/Cu_3_P indicated its superior water dissociation capacity compared to Ti_3_C_2_/Cu.^[^ ^1^, ^50^ ^]^ Notably, for Ti_3_C_2_/Cu_3_P, water dissociation preferentially occurs on the Cu_3_P nanoparticles rather than on the Ti_3_C_2_ support (Figure S35 and Supplementary Note 10). Meanwhile, the enhanced water dissociation does not lead to H_2_ evolution during the EHAE process. The Bode plots obtained from EIS date reflect both electron‐transfer process and their kinetics during electrocatalysis. As shown in Figure 5b, the phase angle peak intensities decreased when the atmosphere was switched from Ar to C_2_H_2_ over Ti_3_C_2_/Cu_3_P, indicating that C_2_H_2_ hydrogenation kinetically outcompetes HER. Notably, under Ar flow, Ti_3_C_2_/Cu_3_P exhibited a higher phase angle intensity and a lower peak frequency compared to Ti_3_C_2_/Cu, suggesting suppressed HER kinetics on Ti_3_C_2_/Cu_3_P (Figure S36).^[^ ^51^ ^]^

Density functional theory (DFT) calculations revealed that the incorporation of P atoms shifted the *d*‐band center of Cu closer to the Fermi level on Ti_3_C_2_/Cu_3_P, providing more empty antibonding *d*‐orbitals for active H^*^ and thus enhancing H^*^ adsorption (Figure 5c).^[^ ^20^, ^52^ ^]^ We further analyzed the free energy profiles for water dissociation and HER on Ti_3_C_2_/Cu_3_P and Ti_3_C_2_/Cu (Table S6). As shown in Figure 5d, Ti_3_C_2_/Cu_3_P exhibited stronger water adsorption (−0.61 eV) and a lower activation energy for water dissociation (0.50 eV) compared to Ti_3_C_2_/Cu (−0.52 eV and 0.79 eV, respectively). These results indicated that Ti_3_C_2_/Cu_3_P efficiently supplied H^*^ and promoted the protonation process during the EHAE process. Moreover, Ti_3_C_2_/Cu_3_P exhibited a higher energy barrier for HER (0.1 eV) compared to Ti_3_C_2_/Cu (−0.04 eV), which highlights its ability to suppress HER (Figure 5e). Raman spectroscopy was performed on Ti_3_C_2_/Cu_3_P before and after EHAE process. The bands at 151 cm^−1^ and 622 cm^−1^ were attributed to the in‐plane and out‐of‐plane Ti─C vibrations of Ti_3_C_2_ with *E*_g_ and *A*_1g_ symmetry, respectively, and these remained unchanged after the reaction (Figure S37).^[^ ^53^, ^54^ ^]^ Additionally, EDS analysis confirmed the uniform distribution of Ti, Cu, P, and C elements after the EHAE reaction (Figure S38).

In situ Raman spectroscopy was employed to identify reaction intermediates during the EHAE process (Figure 5f and Figures S39 and S40). The peaks at 1332 and 1578 cm^−1^ are assigned to the D and G bands of carbon, respectively. Upon applying a cathodic potential from 0 to −0.2 V versus RHE, two new peaks emerged at 1119 and 1500 cm^−1^, which correspond to the C─C and C═C bonds in polyacetylene, respectively. As the potential was further reduced, the intensities of these polyacetylene characteristic peaks increased on both Ti_3_C_2_/Cu_3_P and Ti_3_C_2_/Cu, indicating progressive intensification of the EHAE process.^[^ ^40^, ^55^, ^56^ ^]^ In addition, a broad band between 3100 and 3600 cm^−1^ was monitored and attributed to the O─H stretching of interfacial water. Gaussian deconvolution resolved three hydrogen‐bonding states in this region: 4HB‐H_2_O (3220 cm^−1^), 2HB‐H_2_O (3405 cm^−1^), and cation‐bound water (K^+^‐H_2_O, 3550 cm^−1^), respectively. As the potential became more negative, the intensity of the K^+^‐H_2_O signal increased, indicating accelerated dissociation of interfacial water that correlates with an increased hydrogenation rate.^[^ ^57^, ^58^ ^]^ Notably, the relative fraction of K^+^‐H_2_O was consistently higher on Ti_3_C_2_/Cu_3_P than on Ti_3_C_2_/Cu, suggesting that Ti_3_C_2_/Cu_3_P promotes interfacial water dissociation more effectively—consistent with the EPR results (Figure S41 and Figure 5a).

Regarding the kinetics of the EHAE process, Figure 5g illustrated that the free energy for C_2_H_2_ adsorption on Ti_3_C_2_/Cu and Ti_3_C_2_/Cu_3_P was −0.97 eV and −1.54 eV, respectively, indicating a stronger C_2_H_2_ affinity on Ti_3_C_2_/Cu_3_P. Notably, the adsorption of H^*^on the Ti_3_C_2_/Cu_3_P surface was exothermic (−0.07 eV), whereas it was endothermic (0.068 eV) on Ti_3_C_2_/Cu, suggesting that H^*^ adsorption was energetically more favorable on Ti_3_C_2_/Cu_3_P.^[^ ^9^ ^]^ For the following two hydrogenation steps, the adsorbed C_2_H_2_ was hydrogenated to form C_2_H_4_^*^ with favorable downhill energy profiles. The transformation from C_2_H_4_^*^ + H^*^ to C_2_H_5_^*^ is commonly recognized as the rate‐determining step for the over‐hydrogenation of C_2_H_4_.^[^ ^11^, ^59^ ^]^ For Ti_3_C_2_/Cu_3_P, this step was endothermic (0.42 eV), whereas it was exothermic (−0.64 eV) for Ti_3_C_2_/Cu, indicating that Ti_3_C_2_/Cu tended to over‐hydrogenate C_2_H_4_ (Table S7). These results support a plausible EHAE mechanism on the Ti_3_C_2_/Cu_3_P surface: initially, water and C_2_H_2_ molecules are adsorbed onto the surface, where rapid water dissociation generates abundant active H^*^ that react with the adsorbed C_2_H_2_ to form C_2_H_4_. The resulting C_2_H_4_ is then rapidly desorbed as C_2_H_4_ (g). Owing to its excellent hydrogenation activity and relatively high HER energy barrier, H^*^ is more inclined to participate in the hydrogenation of C_2_H_2_ rather than couple to form H_2_. This behavior leads to superior EHAE performances at industrial current densities (Figure 5h).

## Conclusions

In summary, we report a highly efficient and stable electrocatalyst, Ti_3_C_2_/Cu_3_P, for EHAE conversion. By anchoring Cu_3_P nanoparticles onto the MXene matrix through Ti─O─P bonds, the strong interactions not only facilitated water dissociation but also synergistically enhanced the adsorption of C_2_H_2_ and H^*^, while effectively preventing catalyst dissolution and agglomeration. This enabled both efficient and stable electrochemical C_2_H_4_ synthesis. When assembled as the cathode, Ti_3_C_2_/Cu_3_P achieved a record energy efficiency of 23.0% at 0.2 A cm^−2^. Moreover, it demonstrated continuous operation for over 100 h at 1.0 A in a 4 cm^2^ MEA, outperforming most previously reported electrocatalysts. The breakthrough in full‐cell energy efficiency and durability represents a significant advancement toward achieving environmental sustainability and economic viability in C_2_H_4_ productions.

## Conflict of Interests

The authors declare no conflict of interest.

## Supporting information



Supporting Information

## Data Availability

The data that support the findings of this study are available from the corresponding author upon reasonable request.

## References

[1] L. Huang , D. Bao , Y. Jiang , Y. Zheng , S. Qiao , Angew. Chem. Int. Ed. 2024, 63, e202405943. 10.1002/anie.202405943 38769621

[2] Y. Gao , L. Neal , D. Ding , W. Wu , C. Baroi , A. M. Gaffney , F. Li , ACS Catal. 2019, 9, 8592–8621.

[3] Z. Li , M. Åhman , L. J. Nilsson , F. Bauer , Renew. Sustain. Energy Rev. 2024, 199, 114540.

[4] B.‐H. Zhao , F. Chen , M. Wang , C. Cheng , Y. Wu , C. Liu , Y. Yu , B. Zhang , Nat. Sustain. 2023, 6, 827–837.

[5] S. Wang , K. Uwakwe , L. Yu , J. Ye , Y. Zhu , J. Hu , R. Chen , Z. Zhang , Z. Zhou , J. Li , Z. Xie , D. Deng , Nat. Commun. 2021, 12, 7072. 34873161 10.1038/s41467-021-27372-8 PMC8648715

[6] F. Chen , L. Li , C. Cheng , Y. Yu , B.‐H. Zhao , B. Zhang , Nat. Commun. 2024, 15, 5914. 39003284 10.1038/s41467-024-50335-8 PMC11246534

[7] C. Liu , F. Chen , B.‐H. Zhao , Y. Wu , B. Zhang , Nat. Rev. Chem. 2024, 8, 277–293. 38528116 10.1038/s41570-024-00589-z

[8] J. Bu , Z. Liu , W. Ma , L. Zhang , T. Wang , H. Zhang , Q. Zhang , X. Feng , J. Zhang , Nat. Catal. 2021, 4, 557–564.

[9] L. Zhang , Z. Chen , Z. Liu , J. Bu , W. Ma , C. Yan , R. Bai , J. Lin , Q. Zhang , J. Liu , T. Wang , J. Zhang , Nat. Commun. 2021, 12, 6574. 34772929 10.1038/s41467-021-26853-0 PMC8589958

[10] C. Cheng , F. Chen , B. Zhang , B. Zhao , X. Du , Angew. Chem. Int. Ed. 2025, 64, e202413897. 10.1002/anie.202413897 39271455

[11] R. Shi , Z. Wang , Y. Zhao , G. I. N. Waterhouse , Z. Li , B. Zhang , Z. Sun , C. Xia , H. Wang , T. Zhang , Nat. Catal. 2021, 4, 565–574.

[12] L. Bai , Y. Wang , Z. Han , J. Bai , K. Leng , L. Zheng , Y. Qu , Y. Wu , Nat. Commun. 2023, 14, 8384. 38104169 10.1038/s41467-023-44171-5 PMC10725425

[13] W. Xue , X. Liu , C. Liu , X. Zhang , J. Li , Z. Yang , P. Cui , H.‐J. Peng , Q. Jiang , H. Li , P. Xu , T. Zheng , C. Xia , J. Zeng , Nat. Commun. 2023, 14, 2137. 37059857 10.1038/s41467-023-37821-1 PMC10104804

[14] Z. Wu , J. Zhang , Q. Guan , X. Liu , H. Xiong , S. Chen , W. Hong , D. Li , Y. Lei , S. Deng , J. Wang , G. Wang , Adv. Mater. 2024, 36, 2408681. 10.1002/adma.202408681 39155581

[15] Z. Wu , T. Wang , Q. Guan , D. Li , Y. Lei , Y. Chen , W. Hong , C. Wu , S. K. Sharma , J. Zhang , S. Chen , G. Wang , J. Wang , CCS Chem. 2025, 7, 2844–2853.

[16] Y. Gu , Y. Tan , H. Tan , Y. Han , D. Cheng , F. Lin , Z. Qian , L. Zeng , S. Zhang , R. Zeng , Y. Liu , H. Guo , M. Luo , S. Guo , Nat. Synth. 2025, 4, 614–621.

[17] X. Jiang , L. Tang , L. Dong , X. Sheng , W. Zhang , Z. Liu , J. Shen , H. Jiang , C. Li , Angew. Chem. Int. Ed. 2023, 135, e202307848. 10.1002/anie.202307848 37378584

[18] Z. Wang , C. Li , G. Peng , R. Shi , L. Shang , T. Zhang , Angew. Chem. Int. Ed. 2024, 63, e202400122. 10.1002/anie.202400122 38494445

[19] M. Xu , M. Peng , H. Tang , W. Zhou , B. Qiao , D. Ma , J. Am. Chem. Soc. 2024, 146, 2290–2307. 38236140 10.1021/jacs.3c09102

[20] L. Liu , Q. Zhao , R. Liu , L. Zhu , Appl. Catal. B Environ. 2019, 252, 198–204.

[21] B. R. Anne , J. Kundu , M. K. Kabiraz , J. Kim , D. Cho , S. Choi , Adv. Funct. Mater. 2023, 33, 2306100.

[22] V. Kamysbayev , A. S. Filatov , H. Hu , X. Rui , F. Lagunas , D. Wang , R. F. Klie , D. V. Talapin , Science 2020, 369, 979–983. 32616671 10.1126/science.aba8311

[23] Y. Li , H. Shao , Z. Lin , J. Lu , L. Liu , B. Duployer , P. O. Å. Persson , P. Eklund , L. Hultman , M. Li , K. Chen , X. H. Zha , S. Du , P. Rozier , Z. Chai , E. Raymundo‐Piñero , P. L. Taberna , P. Simon , Q. Huang , Nat. Mater. 2020, 19, 894–899. 32284597 10.1038/s41563-020-0657-0

[24] Y. Zhao , J. Zhang , X. Guo , X. Cao , S. Wang , H. Liu , G. Wang , Chem. Soc. Rev. 2023, 52, 3215–3264. 37073529 10.1039/d2cs00698g

[25] Q. Zhang , J. Wang , Q. Yu , Q. Li , R. Fan , C. Li , Y. Fan , C. Zhao , W. Cheng , P. Ji , J. Sheng , C. Zhang , S. Xie , G. Henkelman , H. Li , Nat. Synth. 2024, 4, 252–261.

[26] Y. Zhang , Q. Zhao , B. Danil , W. Xiao , X. Yang , Adv. Mater. 2024, 36, 2400198. 10.1002/adma.202400198 38452354

[27] L. Liu , S. Zheng , H. Chen , J. Cai , S. Zang , Angew. Chem. Int. Ed. 2024, 63, e202316910. 10.1002/anie.202316910 38179795

[28] Y. Yoon , A. P. Tiwari , M. Choi , T. G. Novak , W. Song , H. Chang , T. Zyung , S. S. Lee , S. Jeon , K. An , Adv. Funct. Mater. 2019, 29, 1903443.

[29] F. O. Boakye , F. uz Zaman , H. Zhang , A. Saeed , F. T. Dajan , S. Iqbal , K. Harrath , Adv. Funct. Mater. 2025, 35, 2424718.

[30] S. Yang , J. Bu , R. Bai , J. Lin , S. An , Y. Wu , Y. Guo , J. Gao , J. Zhang , Chinese J. Chem. 2023, 41, 3618–3624.

[31] Q. Zhao , C. Zhang , R. Hu , Z. Du , J. Gu , Y. Cui , X. Chen , W. Xu , Z. Cheng , S. Li , B. Li , Y. Liu , W. Chen , C. Liu , J. Shang , L. Song , S. Yang , ACS Nano 2021, 15, 4927–4936. 33617242 10.1021/acsnano.0c09755

[32] M. Wang , Z. Wang , Z. Huang , M. Fang , Y. Zhu , L. Jiang , ACS Nano 2024, 18, 15303–15311. 38803281 10.1021/acsnano.4c04780

[33] J. Tian , Q. Liu , N. Cheng , A. M. Asiri , X. Sun , Angew. Chemie Int. Ed. 2014, 53, 9577–9581. 10.1002/anie.201403842 25044801

[34] R. Wang , X. Dong , J. Du , J. Zhao , S. Zang , Adv. Mater. 2018, 30, 1703711. 10.1002/adma.201703711 29266417

[35] Y. Bai , C. Liu , T. Chen , W. Li , S. Zheng , Y. Pi , Y. Luo , H. Pang , Angew. Chem. Int. Ed. 2021, 60, 25318–25322. 10.1002/anie.202112381 34585486

[36] J. Feng , L. Wu , S. Liu , L. Xu , X. Song , L. Zhang , Q. Zhu , X. Kang , X. Sun , B. Han , J. Am. Chem. Soc. 2023, 145, 9857–9866. 37092347 10.1021/jacs.3c02428

[37] X. Zhang , D. Kim , X. Guo , Y. Zhu , L. Y. S. Lee , Appl. Catal. B Environ. 2021, 298, 120515.

[38] J. Zhong , J. Li , Small 2024, 20, 2306241. 10.1002/smll.202306241 37857592

[39] Z. Pan , L. Kang , T. Li , M. Waqar , J. Yang , Q. Gu , X. Liu , Z. Kou , Z. Wang , L. Zheng , J. Wang , ACS Nano 2021, 15, 12975–12987. 34370437 10.1021/acsnano.1c01817

[40] R. Bai , J. Li , J. Lin , Z. Liu , C. Yan , L. Zhang , J. Zhang , CCS Chem. 2023, 5, 200–208.

[41] L. Zhang , R. Bai , J. Lin , J. Bu , Z. Liu , S. An , Z. Wei , J. Zhang , Nat. Chem. 2024, 16, 893–900. 38641678 10.1038/s41557-024-01480-6

[42] C. P. O'Brien , R. K. Miao , A. Shayesteh Zeraati , G. Lee , E. H. Sargent , D. Sinton , Chem. Rev. 2024, 124, 3648–3693. 38518224 10.1021/acs.chemrev.3c00206

[43] J. Chen , H. Qiu , Y. Zhao , H. Yang , L. Fan , Z. Liu , S. Xi , G. Zheng , J. Chen , L. Chen , Y. Liu , L. Guo , L. Wang , Nat. Commun. 2024, 15, 5893. 39003258 10.1038/s41467-024-50269-1 PMC11246503

[44] J. Li , Y. Guo , S. Chang , J. Lin , Y. Wang , Z. Liu , Y. Wu , J. Zhang , Small 2023, 19, 2205845. 10.1002/smll.202205845 36446635

[45] W. Ma , Z. Chen , J. Bu , Z. Liu , J. Li , C. Yan , L. Cheng , L. Zhang , H. Zhang , J. Zhang , T. Wang , J. Zhang , J. Mater. Chem. A 2022, 10, 6122–6128.

[46] Z. Liu , Z. Chen , J. Bu , W. Ma , L. Zhang , H. Zhong , L. Cheng , S. Li , T. Wang , J. Zhang , Chem. Eng. J. 2022, 431, 134129.

[47] X. Gao , D. Wang , R. Bai , D. Lin , J. Zhang , Z. Li , Y. Hou , L. Lei , B. Yang , Adv. Funct. Mater. 2025, 35, 2415384.

[48] C. Yan , Y. Wang , Y. Rong , X. Su , X. Zhang , D. Gao , G. Wang , X. Bao , Angew. Chem. Int. Ed. 2025, e202513162. 10.1002/anie.202513162 40904204

[49] Y. He , F. Chen , B. Zhao , C. Cheng , B. Zhang , Angew. Chem. Int. Ed. 2025, e202518663. 10.1002/anie.202518663 41090191

[50] X. Cao , Y. Ding , D. Chen , W. Ye , W. Yang , L. Sun , J. Am. Chem. Soc. 2024, 146, 25125–25136. 39110104 10.1021/jacs.4c08205

[51] B. Miao , F. Chen , C. Cheng , M. Tao , B. Zhao , B. Zhang , Angew. Chem. Int. Ed. 2025, 64, e202502757. 10.1002/anie.202502757 39996605

[52] Y. Shi , Z.‐R. Ma , Y.‐Y. Xiao , Y.‐C. Yin , W.‐M. Huang , Z.‐C. Huang , Y.‐Z. Zheng , F.‐Y. Mu , R. Huang , G.‐Y. Shi , Y.‐Y. Sun , X.‐H. Xia , W. Chen , Nat. Commun. 2021, 12, 3021. 34021141 10.1038/s41467-021-23306-6 PMC8140142

[53] A. Sengupta , B. V. B. Rao , N. Sharma , S. Parmar , V. Chavan , S. K. Singh , S. Kale , S. Ogale , Nanoscale 2020, 12, 8466–8476. 32242189 10.1039/c9nr10980c

[54] M. Urso , L. Bruno , S. Dattilo , S. C. Carroccio , S. Mirabella , ACS Appl. Mater. Interfaces 2024, 16, 1293–1307. 38134036 10.1021/acsami.3c13470 PMC10788834

[55] M. L. Patterson , M. J. Weaver , J. Phys. Chem. 1985, 89, 1331–1334.

[56] S. Lefrant , L. S. Lichtmann , H. Temkin , D. B. Fitchen , D. C. Miller , G. E. Whitwell , J. M. Burlitch , Solid State Commun. 1979, 29, 191–196.

[57] S. Tang , N. Guo , C. Chen , B. Yao , X. Liu , C. Ma , Q. Liu , S. Ren , C. He , B. Liu , X. Li , Angew. Chem. Int. Ed. 2025, 64, e202510192. 10.1002/anie.202510192 40757870

[58] X. H. Lv , H. Huang , L. T. Cui , Z. Y. Zhou , W. Wu , Y. C. Wang , S. G. Sun , ACS Appl. Mater. Interfaces 2024, 16, 8668–8678. 38344994 10.1021/acsami.3c15925

[59] Z. Chen , C. Cai , T. Wang , J. Phys. Chem. C 2022, 126, 3037–3042.

