# Supplementary material for: Toward Industrial Electrosynthesis of Ethylene: Energy‐Efficient and Stable Acetylene Semi‐Hydrogenation on a Copper Phosphide/MXene Electrocatalyst
Source: Angew Chem Int Ed Engl. 2026 Jan 19;65(9):e18909. doi: 10.1002/anie.202518909 (PMC12930021; doi:10.1002/anie.202518909)
Supplement: Supplementary file 1 — Supporting Information [file ANIE-65-e18909-s001.docx]

**Supporting Information**

**Towards Industrial Electrosynthesis of Ethylene: Energy-Efficient and Stable Acetylene Semi-Hydrogenation on a Copper Phosphide/MXene Electrocatalyst**

*Zeliang Wu*^1,2,4^*, Qihui Guan*^1,4^*, Tao Wang*^1^*, Dongfang Li*^2^*, Ming Lei*^3^*, Wei Hong*^1^*, Shixia Chen*^1^*, Shijian Wang*^2*^*, Guoxiu Wang*^2*^*, Jun Wang*^1*^

Corresponding authors. Email: [guoxiu.wang@uts.edu.au](mailto:guoxiu.wang@uts.edu.au); [shijian.wang@uts.edu.au](mailto:shijian.wang@uts.edu.au); [jwang7@ncu.edu.cn](mailto:jwang7@ncu.edu.cn)

Table of Contents

Figures S1-S42

**Tables S1-S7**

**References**

Supplementary Methods

**Chemicals and Materials**

MAX phase Ti_3_Al_2_C_2_ was purchased from Jilin 11 Technology Co., Ltd. Copper (Ⅱ) chloride (CuCl_2_, 99%), sodium chloride (NaCl, ≥ 99%), and potassium chloride (KCl, ≥ 99%) were purchased from Sigma-Aldrich. Potassium hydroxide (KOH, 99.99%), potassium sulfate (K_2_SO_4_, 99%), nickel chloride hexahydrate (NiCl_2_·6H_2_O, 99.9%), iron chloride hexahydrate (FeCl_3_·6H_2_O, 99%), ammonium persulfate (H_8_N_2_O_8_S_2_, 99%) and sodium hypophosphite monohydrate (NaH_2_PO_2_∙H_2_O, 99.7%) were purchased from Macklin (China). Sulfuric acid (H_2_SO_4_, 98%) was purchased from Xilong Science Co., Ltd. Pure acetylene (99.999%) was supplied by Nanchang Jiang Bamboo Industry Co., Ltd. The carbon paper gas diffusion layer (Sigracet 22 BB), anion exchange membrane (AEM, Fumasep FAB-PK-130, Sustainion RT), Ni foam, and Nafion perfluorinated resin solution (5 wt%) were purchased from Suzhou Sinero Technology Co., Ltd. Ultrapure water (>18.25 MΩ·cm) was used in all experiments. All the chemical reagents were used as received without further purification.

**Catalyst synthesis**

The synthesis of Ti_3_C_2_-Cu was performed with slight modifications to methods reported in existing literature. Briefly, 200 mg Ti_3_AlC_2_ MAX phase powder and 420 mg CuCl_2_ were mixed and ground to homogeneity. Subsequently, 120 mg NaCl and 152 mg KCl were added, followed by further grinding for even mixing. The mixture was placed in an alumina boat and calcined in a tube furnace under an argon atmosphere at a temperature of 750 °C, with a heating rate of 4 °C min^-1^, and maintained for 24 h. The obtained products were repeatedly washed with deionized water and ethanol and then freeze-dried at room temperature to obtain Ti_3_C_2_/Cu powder. The obtained Ti_3_C_2_/Cu products were then washed with 0.1 M ammonium persulfate solution to remove the Cu nanoparticles. The resulting solution was further cleaned five times with deionized water and five times alcohol and filtered with a microfiltration membrane. Finally, the Ti_3_C_2_ powders were dried under freeze-dried at room temperature.

The synthesis of Ti_3_C_2_/Cu_3_P was achieved through the reaction of previously synthesized Ti_3_C_2_/Cu with phosphine under an argon atmosphere. Specifically, 100 mg of Ti_3_C_2_/Cu was positioned downstream in a tube furnace, while 1000 mg of NaH_2_PO_2_∙H_2_O served as the phosphorus source upstream. The process was conducted at a temperature of 300 °C, with a heating rate of 5 °C min^-1^ for a duration of 2 h. The obtained powder was washed with deionized water and ethanol and then freeze-dried at room temperature to yield the Ti_3_C_2_/Cu_3_P. For comparison, the synthesis method of Cu_3_P nanoparticles (Cu_3_P NPs) followed the same procedure, with the only difference being the use of commercial copper nanoparticles as the copper source.

**Materials characterizations**

*In-situ* Raman spectra using a Confocal LabRam HR800 (HORIBA Jobin Yvon) with 532 nm laser; Quasi-*in-situ* electron paramagnetic resonance (EPR) spectra signals of hydrogen radicals were detected on EMXnano (Bruker). X-ray powder diffraction (XRD) patterns via a Bruker D8 with Cu-Kα radiation; Field emission scanning electron microscopy (FESEM) images on a Zeiss Supra 55VP at 10.0 kV; transmission electron microscopy (TEM) and high-resolution TEM (HRTEM) analyses with a Talos F200 at 200 kV; X-ray photoelectron spectroscopy (XPS) data from a Thermo Scientific ESCALAB250Xi; Inductively-coupled plasma mass spectrometry (ICP-MS) date from a Agilent 7800; X-ray absorption fine structure (XAS) spectra collected at the Australian National Synchrotron, processed using ATHENA software.

**Electrode preparation**

A spray-coating strategy after dispersion was employed to prepare the working electrodes. A uniform suspension ink was prepared by dispersing 9 mg of catalyst and 3 mg Ketjenblack in 9 mL of methanol, with the addition of 90 µL of Nafion solution, employing ultrasonic dispersion for 1 hour to ensure thorough mixing. This ink was then meticulously sprayed onto a gas diffusion electrode surface using a spray gun, catalyst mass loading about 0.6 mg cm^-2^. Following the spraying process, the electrode was comprehensively dried to guarantee the complete evaporation of the solvent.

To compare the effects of different counter electrodes on the full cell voltage, we prepared Ni foam and Ni foam loaded with nickel-iron layered double hydroxide (NiFe-LDH/NF) as counter electrodes. The preparation of NiFe-LDH/NF was as follows: a 2.7×2.7 cm piece of Ni foam was first ultrasonically cleaned in ethanol and 5 M HCl, followed by rinsing with acetone and vacuum drying. Subsequently, a solution containing 3 mmol of NiCl_2_·6H_2_O, 2.5 mmol of FeCl_3_·6H_2_O, and 1 mmol of NaCl in a water-ethanol mixture (3:1 ratio) was prepared. The nickel foam was immersed in this solution and left to stand for 18 hours. After the reaction, the foam was thoroughly rinsed with water and ethanol and then dried at 60 ℃ overnight.

**Electrochemical measurements**

**Flow cell:** Electrochemical experiments were conducted using a CHI 660E workstation with a CHI 680D high-current amplifier. The three-electrode configuration included a newly prepared electrode for electrocatalysis as the working electrode, complemented by a Hg/HgO electrode and Ni foam or NiFe-LDH/NF as the reference and counter electrodes, respectively. These components were partitioned by a FAB-PK-130 anion exchange membrane to ensure effective separation of the cathode and anode spaces. For the semi-hydrogenation reactions of C_2_H_2_, the gas was supplied at a rate of 20 mL min^-1^, unless specified otherwise. Similarly, all electrolytic procedures were carried out using a 1.0 M KOH solution, unless otherwise indicated. Utilizing the chronopotentiometry (CP) for the electrochemical semihydrogenation of C_2_H_2_ across various current densities for a consistent 20 mins duration ensured the establishment of stable and reliable performance parameters essential for subsequent quantitative analysis.

To ensure a standardized reference frame for potential measurements, values were adjusted to the Reversible Hydrogen Electrode (RHE) scale utilizing the formula:

$$\text{E}_{\text{RHE}}\text{ = }\text{E}_{\text{Hg/HgO}}\text{ }\text{+}\text{ }\text{0.098 V + 0.059 × pH}$$

**Zero-gap MEA reactor:** The cathode and anode were cut to the required sizes (1×1 cm^2^ and 2×2 cm^2^) and pre-soaked in 1.0 M KOH overnight, followed by rinsing with deionized water before use. The Sustainion RT anion exchange membrane was pre-soaked in the electrolyte. After MEA assembly, 25 mL min^-1^ of 1.0 M KOH was fed to the anode and 20 mL min^-1^ of dry C_2_H_2_ to the cathode. Electrochemical testing was performed once the open-circuit voltage stabilized. For the stability test, fresh electrolyte was replenished every 20 h.

**Products analysis**

Gas products were analyzed using online gas chromatography (GC, Agilent 7890 B), where H_2_ was detected with a Haysep Q column and a TCD detector, while C_2_H_4_, C_2_H_2_, C_2_H_6_, and C_4_ were examined using an Alumina column coupled with an FID detector. Quantification of the gas-phase products was performed via the external standard method. The faradaic efficiency (FE) of the gas products was determined according to the following equation:

$$\text{FE}\text{ (\%)}=\frac{n\times C\times v\times F}{V\times I}\times\text{100\%}$$

Where $n$ represents the number of electrons transferred, $C$ denotes the concentration of gaseous products, $v$ is the gas flow rate in milliliters per minute (mL min^-1^), $F$ stands for the Faraday constant, $V$ indicates the molar volume of gas, and $I$ is the total electrical current involved in the reaction.

The energy efficiency denotes the fraction of energy stored in the desired product relative to the total energy supplied to the electrolysis system. Fundamentally, full-cell energy efficiency strongly depends on cell voltage and FE, as described by^[1,2]^:

$$\text{Energy efficiency (\%)}=\frac{E_{eq}}{E_{cell}}\times{FE}_{C_{2}H_{4}}$$

Where $E_{eq}$ is the thermodynamic equilibrium cell potential (V), $E_{cell}$ is the full cell voltage (V) without *iR* correction, and ${FE}_{C_{2}H_{4}}$ is Faradaic efficiency for C_2_H_4_.

Under standard basic conditions, the cathode and anode reactions from EHAE process is described as follow:

Cathode: $C_{2}H_{2\left( g \right)}+2H_{2}O_{\left( l \right)}+2e^{-}\to C_{2}H_{4\left( g \right)}+2{OH^{-}}_{\left( aq \right)}$

Anode: $2{OH^{-}}_{\left( aq \right)}\to\frac{1}{2}O_{2\left( g \right)}+2e^{-}+H_{2}O_{\left( l \right)}$

Overall: $C_{2}H_{2\left( g \right)}+H_{2}O_{\left( l \right)}\to C_{2}H_{4\left( g \right)}+\frac{1}{2}O_{2\left( g \right)}$

The standard Gibbs free energies of formation at 25 ℃ and 1 atm as such:

$\Delta G_{C_{2}H_{2\left( g \right)}}=+209.2 kJ {mol}^{-1}$

$\Delta G_{C_{2}H_{4\left( g \right)}}=+68.1 kJ {mol}^{-1}$

$\Delta G_{H_{2}O_{\left( l \right)}}=-237.1 kJ {mol}^{-1}$

$\Delta G_{{OH^{-}}_{\left( aq \right)}}=-157.3 kJ {mol}^{-1}$

$\Delta G_{O_{2\left( g \right)}}=0 kJ {mol}^{-1}$

So, the $\Delta G_{EHAE}=68.1-\left( 209.2+\left( -237.1 \right) \right)=96 kJ {mol}^{-1}$

$\Delta G_{EHAE}=-nFE_{eq}$, where $n$ is the number of electrons utilized for EHAE process.

$E_{eq}=\frac{96 \times1000}{-2\times96485}=-0.497 V$

The conversion of C_2_H_2_ was calculated according to the following equation:

$$Conversion \left( \% \right)=\frac{C_{feed}-C_{out}}{C_{feed}}\times100\%$$

Where $C_{feed}$ is the C_2_H_2_ concentration in the feed gas and $C_{out}$ is the C_2_H_2_ concentration in the outlet gas.

The selectivity of product C_2_H_4_ was calculated according to the following equation:

$$Selectivity \left( \% \right)=\frac{C_{feed}-C_{out}}{C_{feed}-C_{out}+C_{C_{2}H_{6}+}{2C}_{C_{4}}}\times100\%$$

Where $C_{C_{2}H_{6}}$ and $C_{C_{4}}$ are the concentration of C_2_H_6_ and C_4_ in the outlet gas.

The formation rate of different products was calculated according to the following equation:

$$Formation rate (mmol {mg}^{-1}h^{-1})=\frac{Q\times FE}{n\times F\times t\times m}$$

Where $Q$ is the total charge number, $t$ is the electrolysis time and $m$ is the mass of the catalyst over the electrode.

**Computational methods**

All the DFT calculations are performed by the Vienna Ab initio Simulation Package (VASP) with the projector augmented wave (PAW) method. The exchange-functional is treated using the generalized gradient approximation (GGA) with Perdew-Burke-Ernzerhof (PBE) functional. The energy cutoff for the plane wave basis expansion was set to 400 eV. Partial occupancies of the Kohn-Sham orbitals were allowed using the Gaussian smearing method and a width of 0.2 eV. The structure of Ti_3_C_2_/Cu and Ti_3_C_2_/Cu_3_P were optimized using the k-point of 3×3×1. The self-consistent calculations apply a convergence energy threshold of 10^-5^ eV, and the force convergency was set to 0.05 eV/Å. The transition state was located using the constrained optimization, where the force convergency was set to 0.05 eV/Å.

The free energy corrections were considered at the temperature of 298 K, the following:

$$\text{∆}\text{G = }\text{∆}\text{E + }{\text{∆}\text{G}}_{\text{ZPE}}\text{ + }{\text{∆}\text{G}}_{\text{U}}\text{ }\text{–}\text{ T}\text{∆}\text{S }$$

where ΔE, ΔG_ZPE_, ΔG_U_, and ΔS refer to the DFT calculated energy change, the correction from zero-point energy, the correction from inner energy, and the correction from entropy.

**Supplementary Note 1:** **Techno-economic analysis**

We conducted a techno-economic analysis to examine the economic feasibility of electrosynthesis C_2_H_4_ using C_2_H_2_ from coal arc plasma under optimistic case assumptions. We established techno-economic model parameters based on experimental data and previous reports.

**Input parameters in production for TEA:**

| Input Costs | Value | Ref |
| --- | --- | --- |
| Coal ($ ton^-1^) | 139.3 | ^[3]^ |
| Hydrogen ($ ton^-1^) | 2000 | ^[3]^ |
| Electricity ($ kWh^-1^) | 0.033 | ^[4]^ |
| Water ($ gal^-1^) | 0.0054 | ^[5]^ |
| Membran ($ m^-2^) | 190 | ^[6]^ |
| Copper ($ g^-1^) | 0.0078 | ^[6]^ |
| Nickel (($ g^-1^)) | 0.00249 | ^[6]^ |
| MEA reactor performance | | |
| Current Density (A cm^-2^) | 0.2 | Our work |
| Full-cell Voltage (V) | 2.04 | Our work |
| C_2_H_4_ Faraday Efficiency (%) | 95 | Our work |
| C_4_ Faraday Efficiency (%) | 4 | Our work |
| Hydrogen Faraday Efficiency (%) | 1 | Our work |
| C_2_H_2_ Conversion (%) | 6.7 | Our work |
| Reactor parameters | | |
| Capital Cost of Reactor for C_2_H_2_ Synthesis ($) | 154,7987 | ^[3]^ |
| Reactor Power for C_2_H_2_ Synthesis (kW) | 1000 | ^[3]^ |
| Energy Consumption per ton C_2_H_2_ (kWh) | 14,000 | ^[3]^ |
| Ratio of Hydrogen to Coal | 1:12 | ^[3]^ |
| Stack Cost for Electrolyzer ($ kW^-1^) | 230 | ^[7]^ |
| Installation Factor | 1.12 | ^[8]^ |
| Plant parameters | | |
| C_2_H_4_ Production Rate (tons day^-1^) | 100 | Our work |
| Plant Life (year) | 20 | ^[7]^ |
| Operating Time (days year^-1^) | 360 | ^[9]^ |
| Balance of Plant, BOP | 54% of electrolyzer cost | ^[9]^ |
| Maintenance Cost | 2.5% of capital cost | ^[5]^ |

The total cost of producing C_2_H_2_ through the arc pyrolysis process consisted capital costs, material (coal, hydrogen) costs, and operating (electricity, separation) costs. Separation cost was set at 30% of the cost of electricity.

$Capital cost\mathbf{=}1547987 \$\times\frac{1}{20 year}\times\frac{year}{360}\times\frac{14000 kWh}{1000 kW\times24h}=125.4 \$ {ton}^{-1}$

$Coal cost=139.3 \$ {ton}^{-1}\times\frac{12}{13}=128.58 \$ {ton}^{-1}$

$Hydrogen cost=2000 \$ {ton}^{-1}\times\frac{1}{13}=153.85 \$ {ton}^{-1}$

$Electricity cost=14000 kWh\times\frac{0.033\$}{kWh}=462 \$ {ton}^{-1}$

$Separation cost=462 \$ {ton}^{-1}\times0.3=138.6 \$ {ton}^{-1}$

$\boldsymbol{Total cost}=125.4+128.58+153.85+462+138.6=1008.43 \$ {ton}^{-1}$

The necessary parameters for calculating costs are specified as follows, including the electrolyzer cost per area, total current, electrolyzer area, daily required power, C_2_H_2_ outlet flow rates, as well as the outlet flow rates of various products (C_2_H_4_, C_4_ and H_2_).

$Electrolyzer cost per area=230 \$ {kW}^{-1}\times\frac{0.2 A}{{cm}^{2}}\times2.04 V\times\frac{{10}^{4} {cm}^{2}}{m^{2}}\times\frac{kW}{1,000}\times1.12=1,051.01 \$ m^{-2}$

$Total current=\frac{100,000kg}{day}\times\frac{day}{86,400s}\times\frac{1,000g}{kg}\times\frac{mol}{28g}\times2e^{-}\times\frac{96,485 C}{mol}\times\frac{1}{0.95}=8,396,425.09 A$

$Electrolyzer area=8,396,425.09 A\times\frac{1}{{0.2 A cm}^{-2}}\times\frac{m^{2}}{10,000 {cm}^{2}}=4,198.21 m^{2}$

$Power=2.04 V\times8,396,425.09 A\times\frac{W}{{10}^{6} MW}=17.13 MW$

$C_{2}H_{2} outlet flow rate=8,396,425.09 A\times\frac{1}{2e^{-}}\times\frac{1}{96,485 C {mol}^{-1}}\times\frac{0.026 kg}{mol}\times\frac{86,400 s}{day}\times\frac{1}{0.067}\times\left( 1-0.067 \right)\times\frac{m^{3}}{1.17 kg}\times\frac{day}{24 h}=48,473.17 m^{3}h^{-1}$

$C_{2}H_{4} outlet flow rate=100,000 kg {day}^{-1}\times\frac{m^{3}}{1.25 kg}\times\frac{day}{24 h}=3333.33m^{3}h^{-1}$

$H_{2} outlet flow rate=8,396,425.09A\times0.01\times\frac{1}{2e^{-}}\times\frac{1}{96,485 C {mol}^{-1}}\times\frac{0.002 kg}{mol}\times\frac{86,400s}{day}\times\frac{m^{3}}{0.089 kg}\times\frac{day}{24 h}=35.20m^{3}h^{-1}$

$C_{4} outlet flow rate=8,396,425.09A\times0.04\times\frac{1}{2e^{-}}\times\frac{1}{96,485 C {mol}^{-1}}\times\frac{0.054 kg}{mol}\times\frac{86,400s}{day}\times\frac{m^{3}}{620 kg}\times\frac{day}{24 h}=0.55m^{3}h^{-1}$

$Total outlet flow rate=\left( 4,8473.17+3333.33+35.20+0.55 \right) m^{3}h^{-1}=51,842.25 m^{3}h^{-1}$

The total cost of producing C_2_H_4_ through the EHAE process consisted capital (electrolyzer, BOP and separation equipment) costs, material (C_2_H_2_, water, catalyst and Membrane) costs, operating (electricity, maintenance and separation) costs.

$Electrolyzer=4,198.21 m^{2}\times1,051.01 \$ m^{2}\times\frac{1}{20 year}\times\frac{year}{360 day}\times\frac{day}{100 ton}=6.13 \$ {ton}^{-1}$

$$BOP=6.13 \$ {ton}^{-1}\times\frac{0.35}{0.65}=3.30 \$ {ton}^{-1}$$

$Separation equipment=1989043 \$\times\left( \frac{51,842.25m^{3}h^{-1}}{1000 m^{3}h^{-1}} \right)^{0.7}\times\frac{1}{20 year}\times\frac{year}{360 day}\times\frac{day}{100 ton}=43.81 \$ {ton}^{-1}$

$$C_{2}H_{2}=1008.43 \$ {ton}^{-1}$$

$Water=8,396,425.09 A\times\frac{1}{{4e}^{-}}\times\frac{1}{96,485 C {mol}^{-1}}\times\frac{0.018 kg}{mol}\times\frac{86,400s}{day}\times\frac{0.2642gal}{kg}\times\frac{0.0054\$}{gal}\times\frac{day}{100 ton}=0.48 \$ {ton}^{-1}$

$Membrane=190 \$ m^{-2}\times4198.21 m^{2}\times2\frac{replacement}{year}\times\frac{year}{360 day}\times\frac{day}{100 ton}=44.3 \$ {ton}^{-1}$

$Cu catalyst=0.0078 \$ g^{-1}\times\frac{0.6 mg}{{cm}^{2}}\times\frac{g}{1000 mg}\times\frac{10000 {cm}^{2}}{m^{2}}\times4198.21 m^{2}\times2\frac{replacement}{year}\times\frac{year}{360 day}\times\frac{day}{100 ton}=0.0109 \$ {ton}^{-1}$

$Ni catalyst=0.00249 \$ g^{-1}\times\frac{0.6 mg}{{cm}^{2}}\times\frac{g}{1000 mg}\times\frac{10000 {cm}^{2}}{m^{2}}\times4198.21 m^{2}\times2\frac{replacement}{year}\times\frac{year}{360 day}\times\frac{day}{100 ton}=0.0035 \$ {ton}^{-1}$

$Electricity=17.13 MW\times\frac{24h}{day}\times\frac{33\$}{MWh}\times\frac{day}{100 ton}=135.66 \$ {ton}^{-1}$

$Maintenance=4,198.21 m^{2}\times1,051.01 \$ m^{2}\times0.025\times\frac{year}{360 day}\times\frac{day}{100 ton}=3.06 \$ {ton}^{-1}$

$Separation=51,842.25 m^{3}h^{-1}\times\frac{0.25 kWh}{m^{3}}\times\frac{0.033\$}{kWh}\times\frac{24 h}{day}\times\frac{day}{100 ton}=102.64 \$ {ton}^{-1}$

$$\boldsymbol{Total cost}=6.12+3.30+43.81+1008.43+0.48+44.3+0.0109+0.0035+135.66+3.06+102.64=1347.83 \$ {ton}^{-1}$$

Figures S1-S30


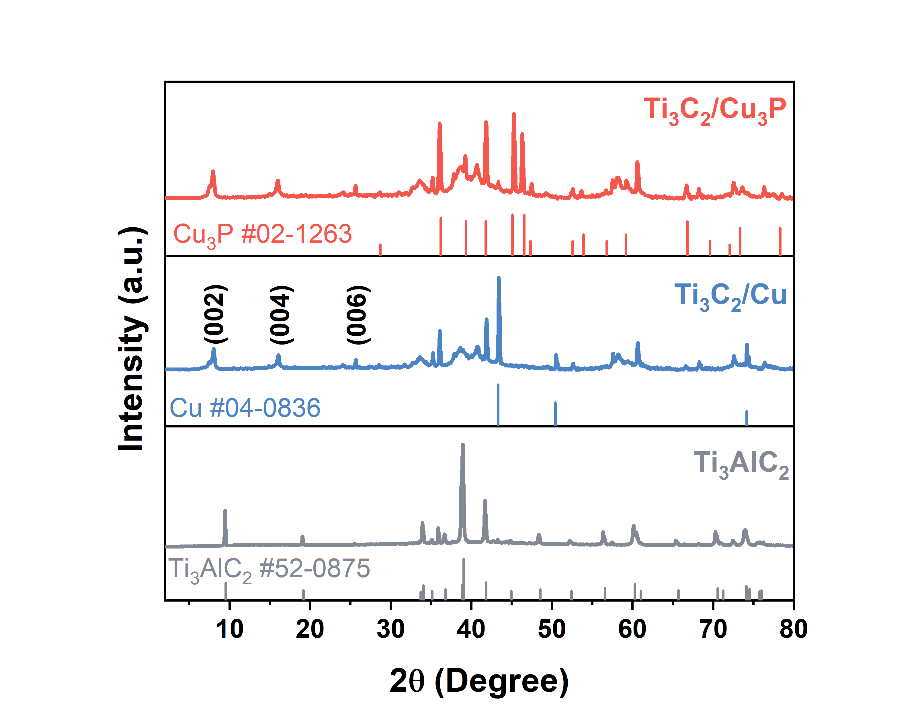


**Figure S1**. XRD patterns of Ti_3_AlC_2_, Ti_3_C_2_/Cu, and Ti_3_C_2_/Cu_3_P.

**Supplementary Note 2**. Compared to the pristine Ti_3_AlC_2_, the (002) peaks in Ti_3_C_2_/Cu shifted from 9.43° to 8.03°, indicating the expansion of interlayer distance from 9.37 Å to over 11.0 Å^[10,11]^. The peaks at 43.3°, 50.5°, and 74.2° correspond to the (111), (200), and (211) planes of metallic Cu (PDF#04-0836), respectively. After phosphidation, the diffraction peaks of Cu disappeared in Ti_3_C_2_/Cu_3_P and were replaced by the characteristic peaks of Cu_3_P (PDF#02-1263).


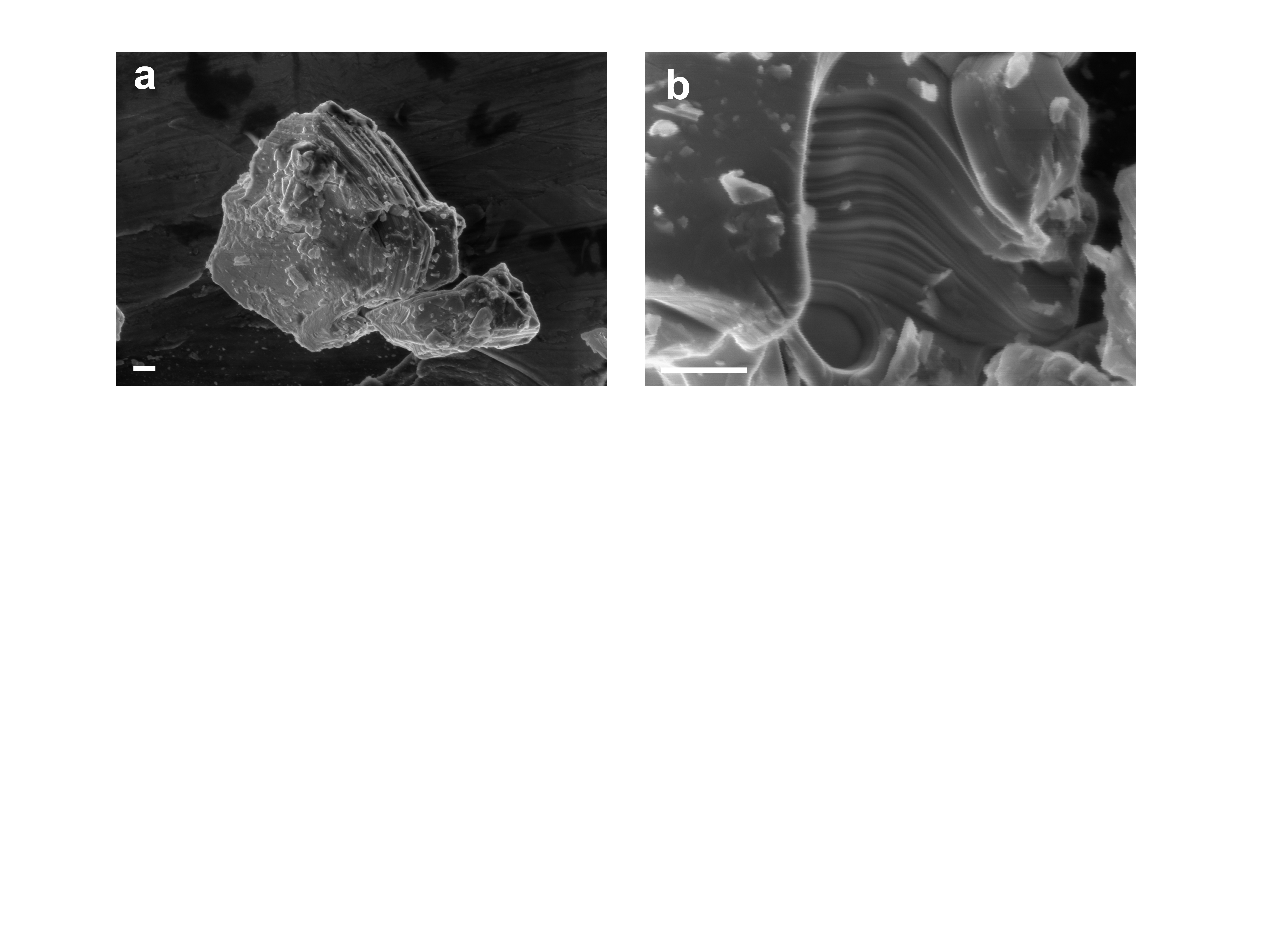


**Figure S2**. SEM images of pristine Ti_3_AlC_2_, scale bar: 1 μm.


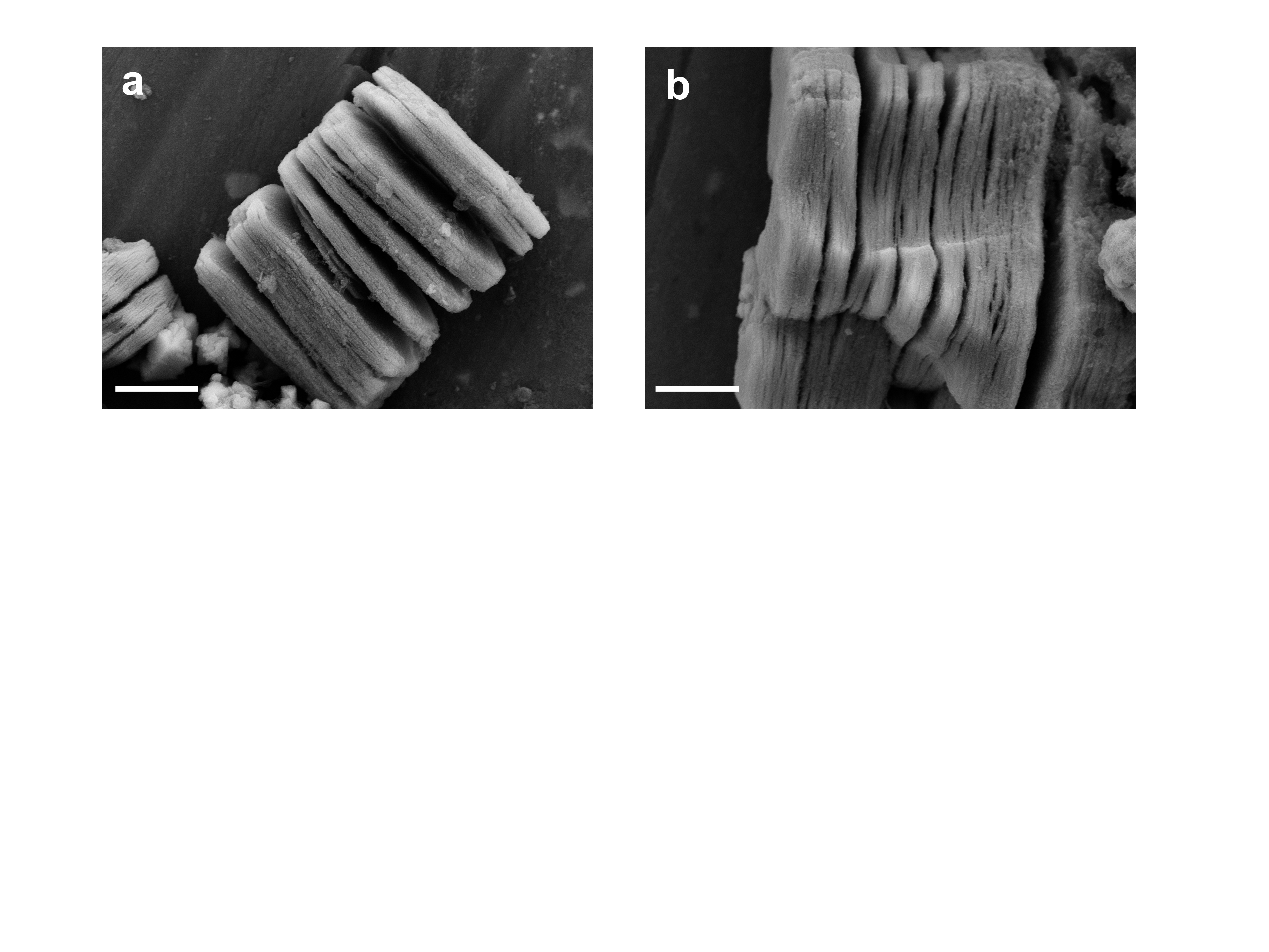


**Figure S3**. SEM images of pristine Ti_3_C_2_/Cu, scale bar: 2 μm.


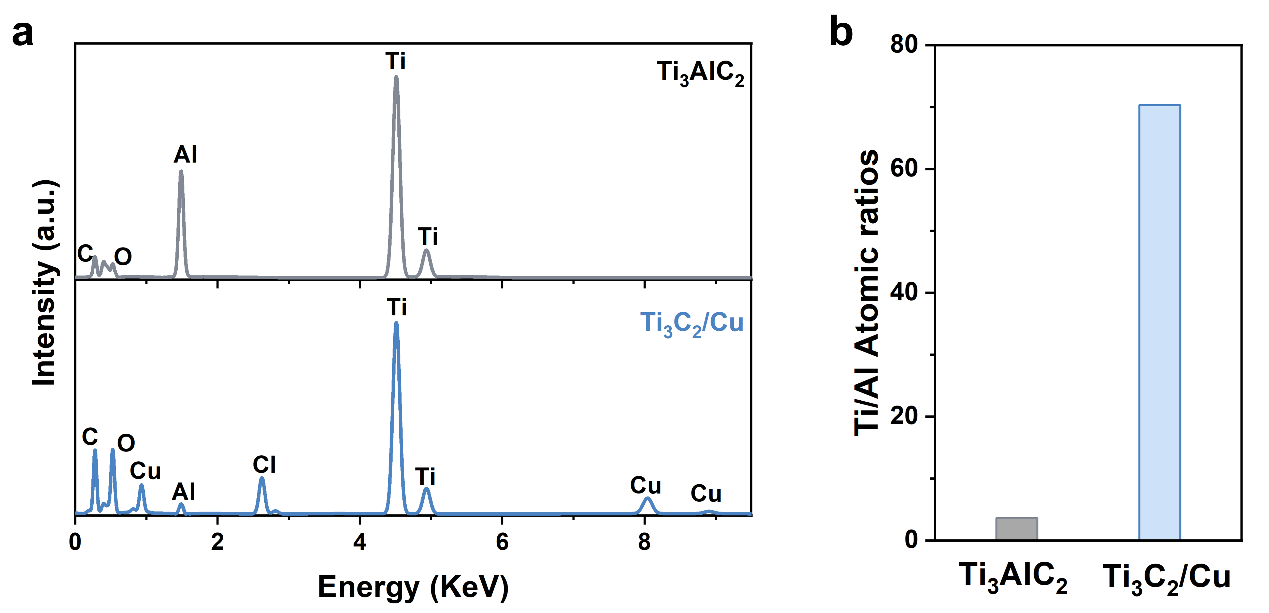


**Figure S4**. (a) EDS analysis of Ti_3_AlC_2_ and Ti_3_C_2_/Cu; (b) Ti/Al Atomic ratios before and after etching.

**Supplementary Note 3**. Compared to pristine Ti_3_AlC_2_, the Al content in Ti_3_C_2_/Cu is substantially decreased, accompanied by a distinct Cu signal. As shown in Figure S4b, the Ti/Al atomic ratio increases sharply after etching, indicating effective substitution of Al by Cu.


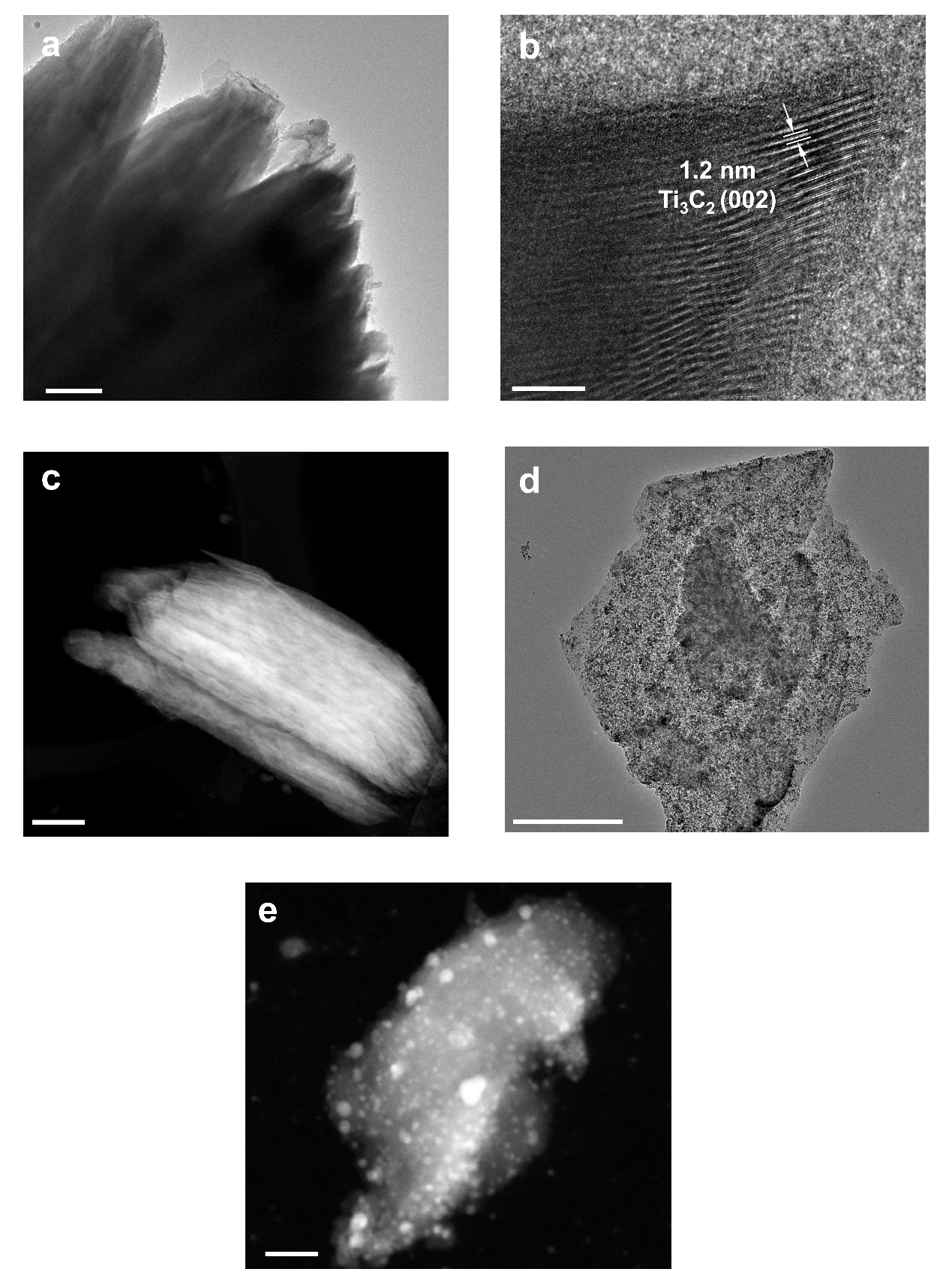


**Figure S5.** TEM and HAADF-STEM images of Ti_3_C_2_/Cu before (**a**, scale bars: 200 nm. **b**, scale bars: 10 nm. **c,** scale bars: 500 nm) and after (**d**, scale bars: 500 nm. **e**, scale bars: 20 nm) ultrasonic peeling.


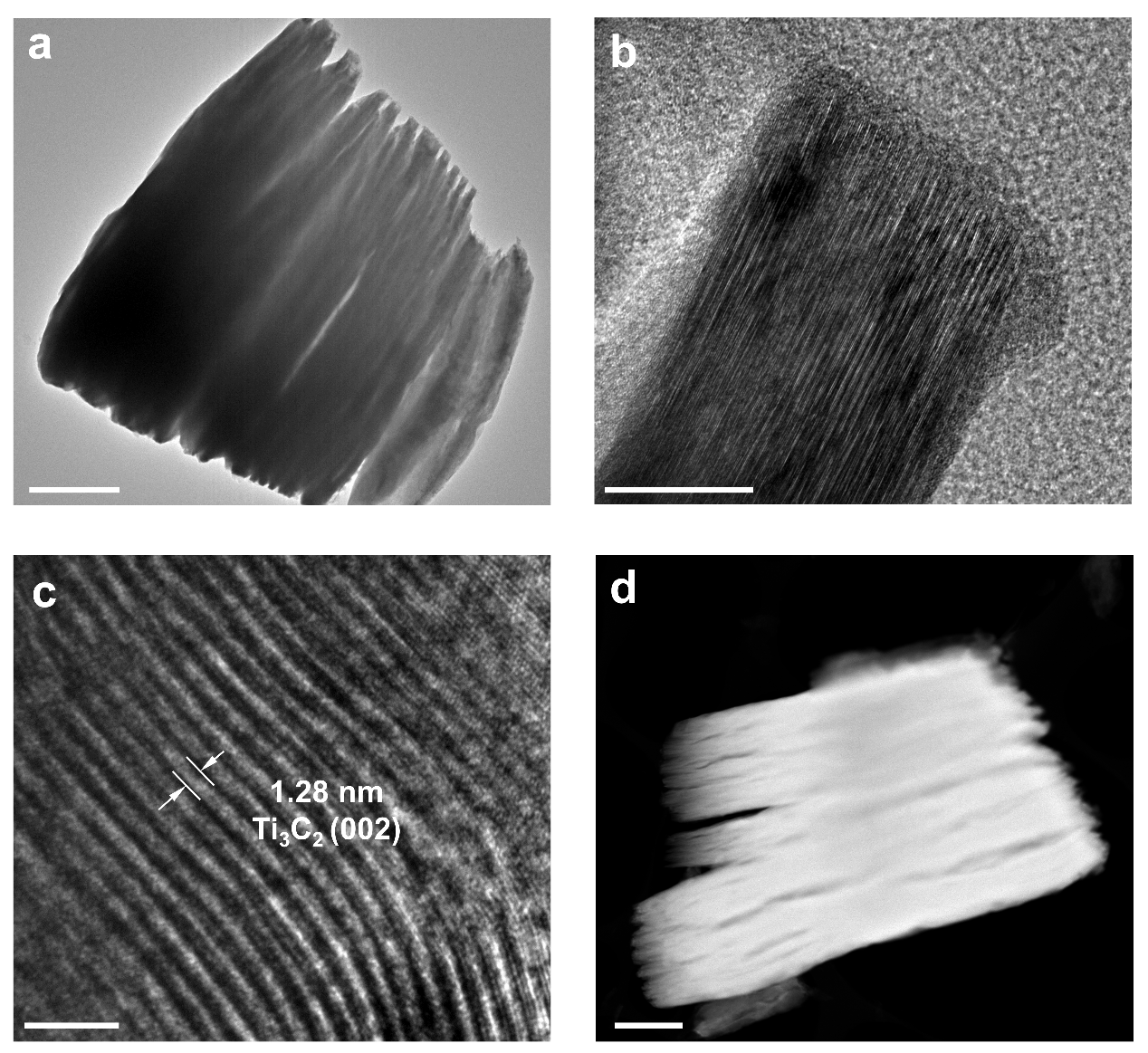


**Figure S6.** TEM (**a**, scale bars: 500 nm. **b**, scale bars: 20 nm. **c**, scale bars: 5 nm) and HAADF-STEM (**d**, scale bars: 1 μm) images of Ti_3_C_2_/Cu_3_P.


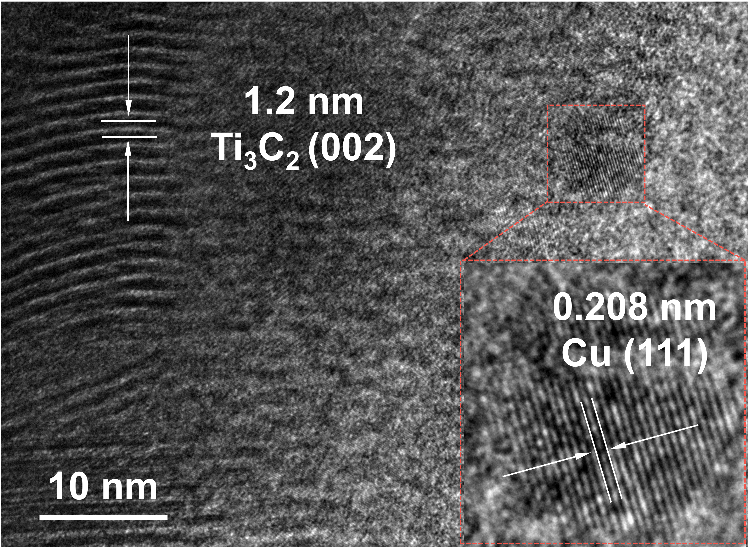


**Figure S7.** HRTEM image of Ti_3_C_2_/Cu. Inset: HRTEM image of Cu nanoparticle in Ti_3_C_2_/Cu.


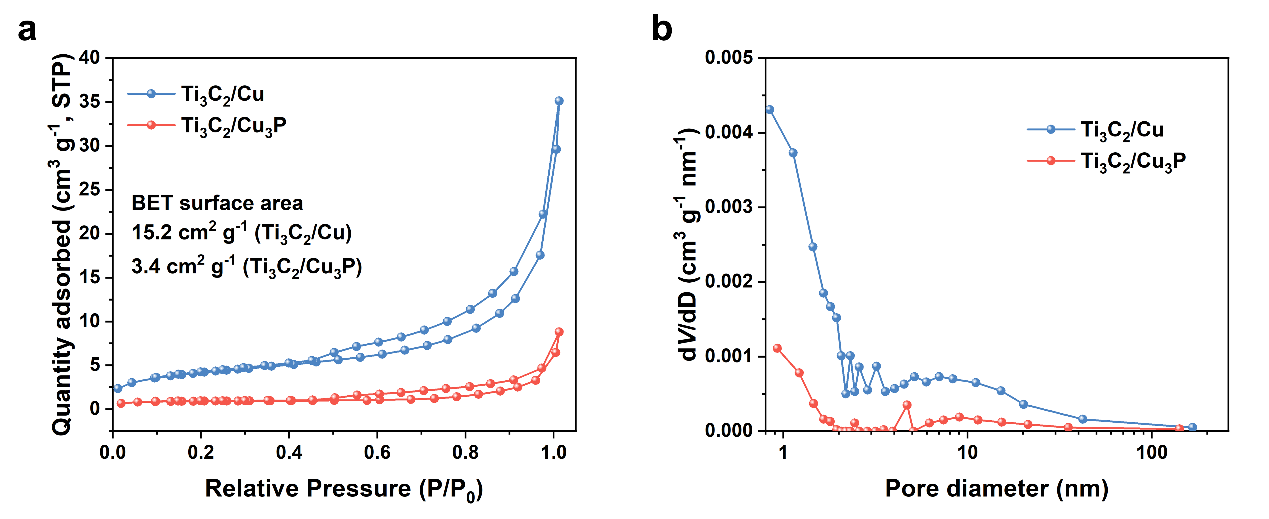


**Figure S8.** (a) Nitrogen adsorption-desorption isotherms and (b) pore diameter distribution curves of Ti_3_C_2_/Cu and Ti_3_C_2_/Cu_3_P.


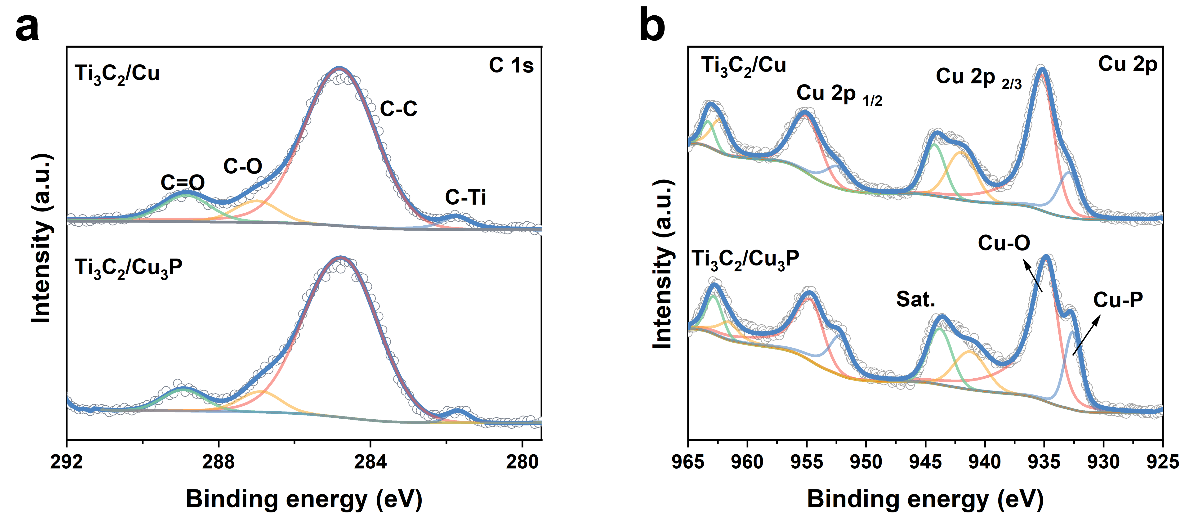


**Figure S9.** High-resolution spectra of C 1s (**a**) and Cu 2p (**b**) for Ti_3_C_2_/Cu and Ti_3_C_2_/Cu_3_P.

**Supplementary Note 4**. The peaks in the C 1s spectra at 281.8, 284.8, 286.8, and 288.9 eV were assigned to Ti-C, C-C, C-O, and C=O bonds, respectively.


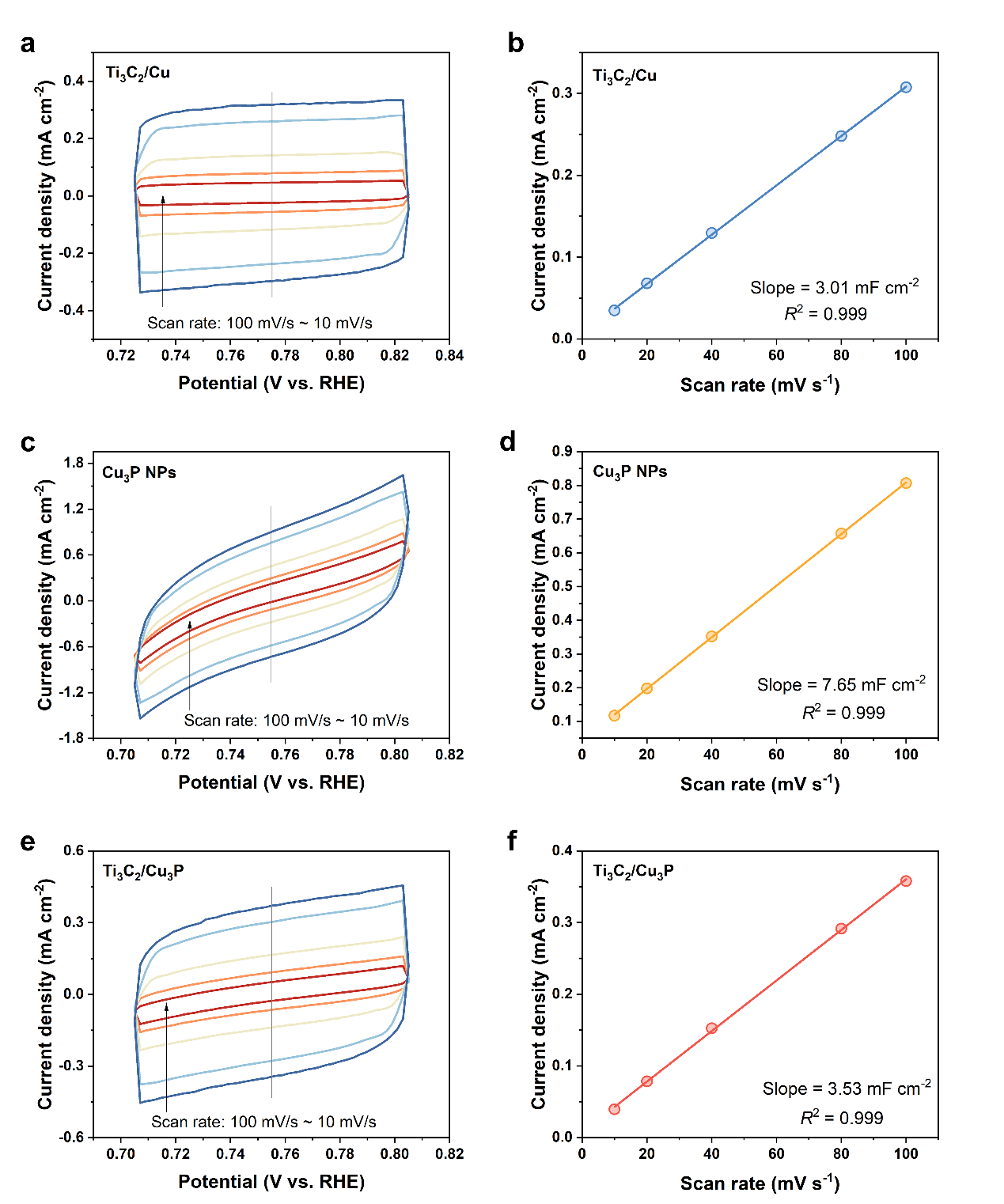


**Figure S10**. Cyclic voltammogram curves at various scan rates and the corresponding fitting for (a, b) Ti_3_C_2_/Cu, (c, d) Cu_3_P NPs, and (e, f) Ti_3_C_2_/Cu_3_P.


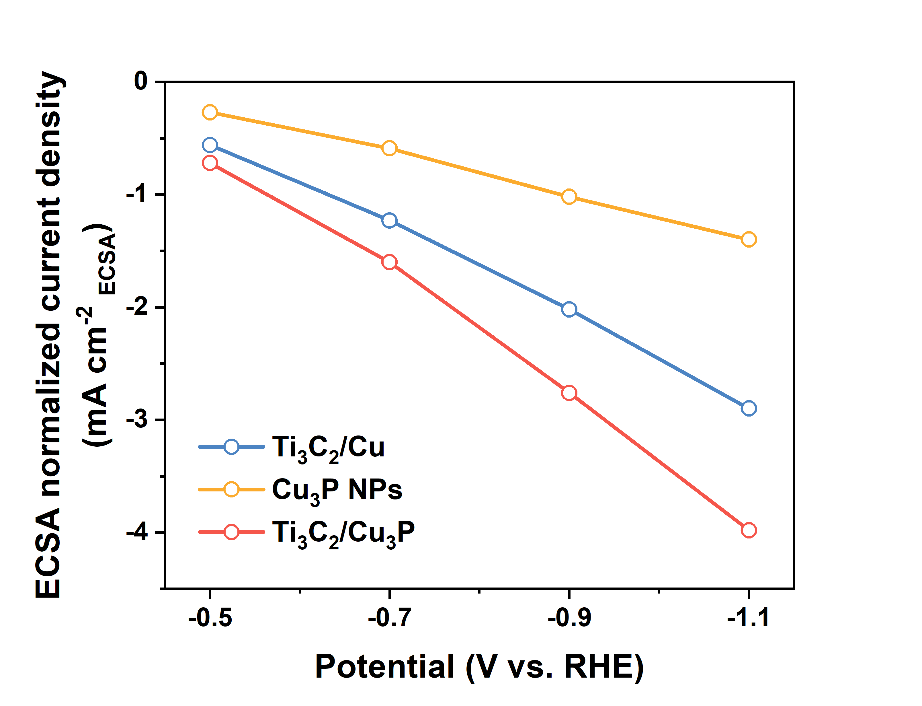


**Figure S11.** The current density of Ti_3_C_2_/Cu, Cu_3_P NPs, and Ti_3_C_2_/Cu_3_P after normalization to the ECSA. Note: In the ECSA calculations, a specific capacitance value of 40 µF cm^-2^ was assumed.


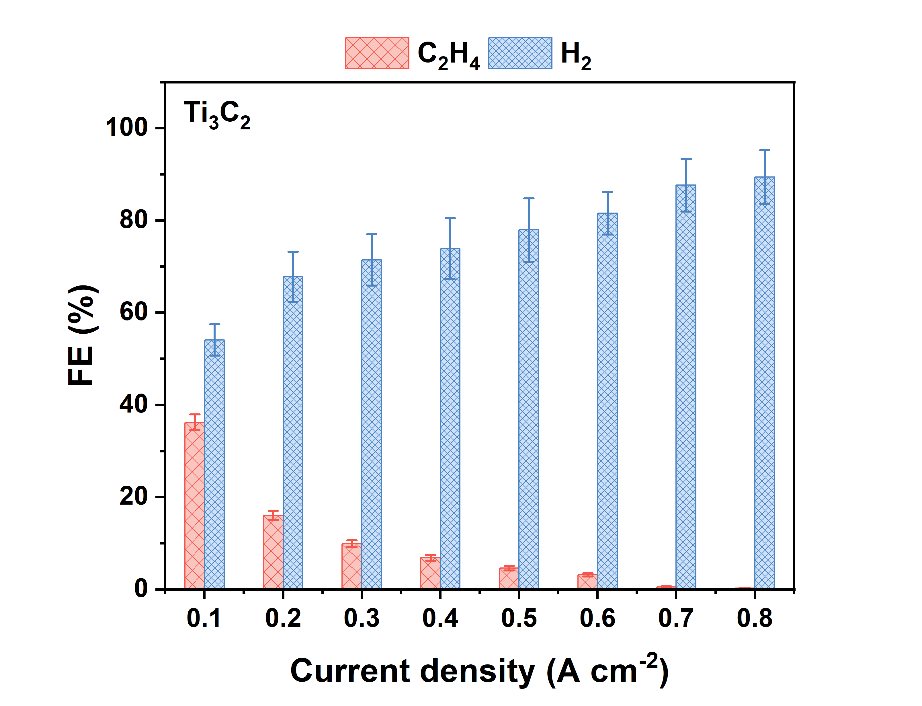


**Figure S12.** The FE of EHAE products at different current densities of Ti_3_C_2_.


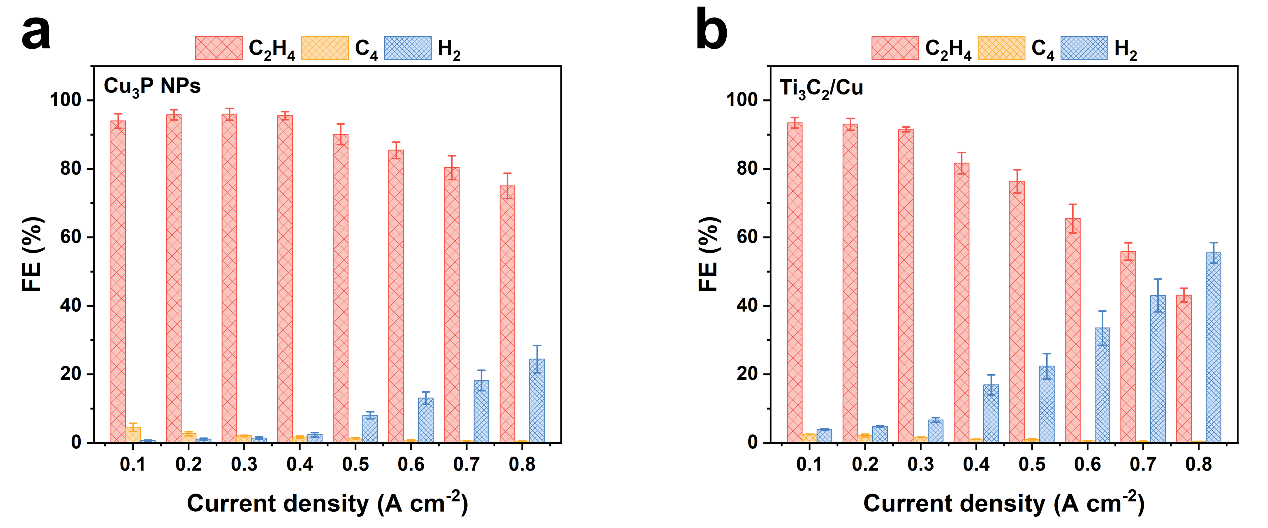


**Figure S13.** The FE of EHAE products at different current densities for (**a**) Cu_3_P NPs and (**b**) Ti_3_C_2_/Cu.


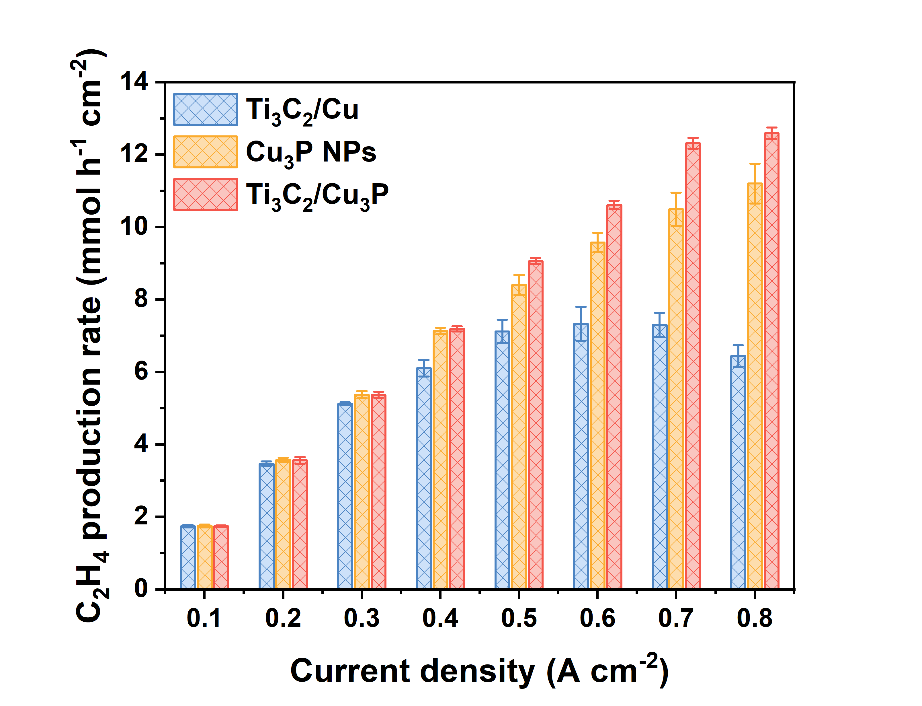


**Figure S14**. The production rate of C_2_H_4_ for Ti_3_C_2_/Cu, Cu_3_P NPs, and Ti_3_C_2_/Cu_3_P at different current densities.


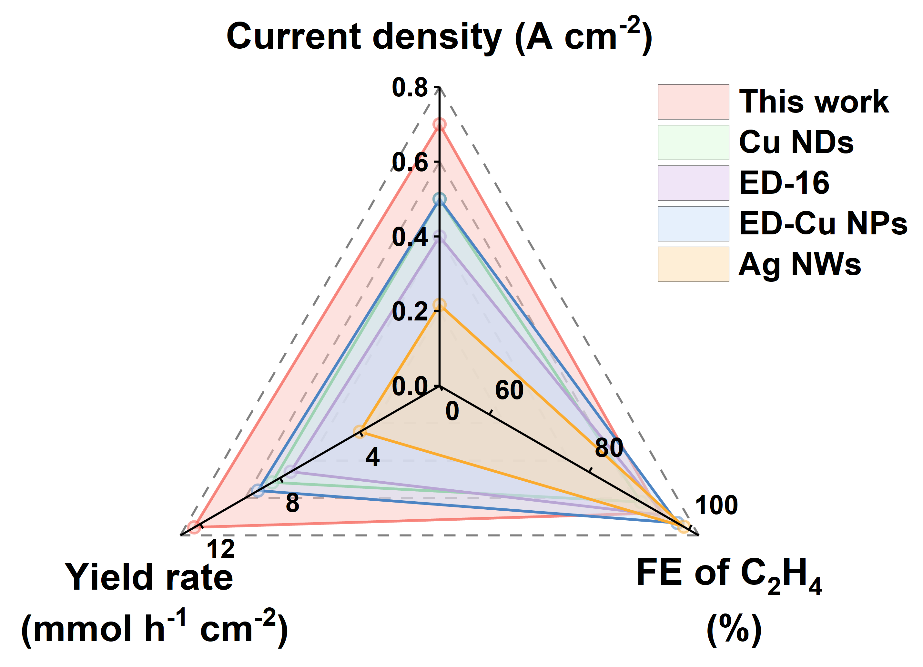


**Figure S15**. Performance comparison of Ti_3_C_2_/Cu_3_P with previously reported catalysts.


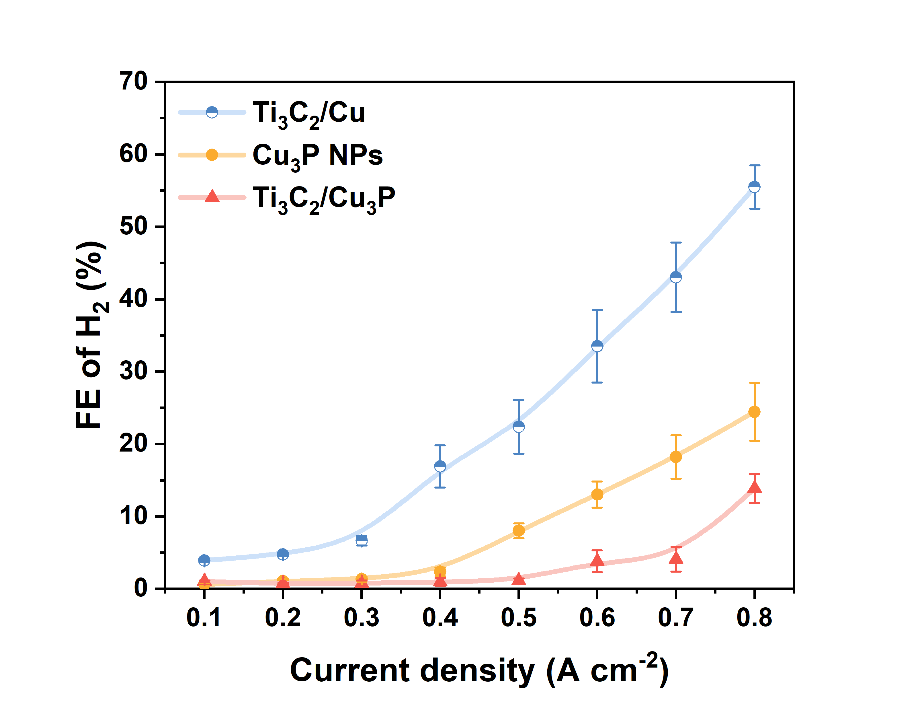


**Figure S16**. The FE of H_2_ at different current densities of Ti_3_C_2_/Cu_3_P, Cu_3_P NPs, and Ti_3_C_2_/Cu.

**Supplementary Note 5**. As the current density increased, the FE of H_2_ gradually increased. At 0.8 A cm^-2^, the H_2_ FE of Ti_3_C_2_/Cu_3_P was only 13.9%, lower than that of Cu_3_P NPs (24.4%) and Ti_3_C_2_/Cu (55.5%), indicating that Ti_3_C_2_/Cu_3_P effectively suppressed the HER.


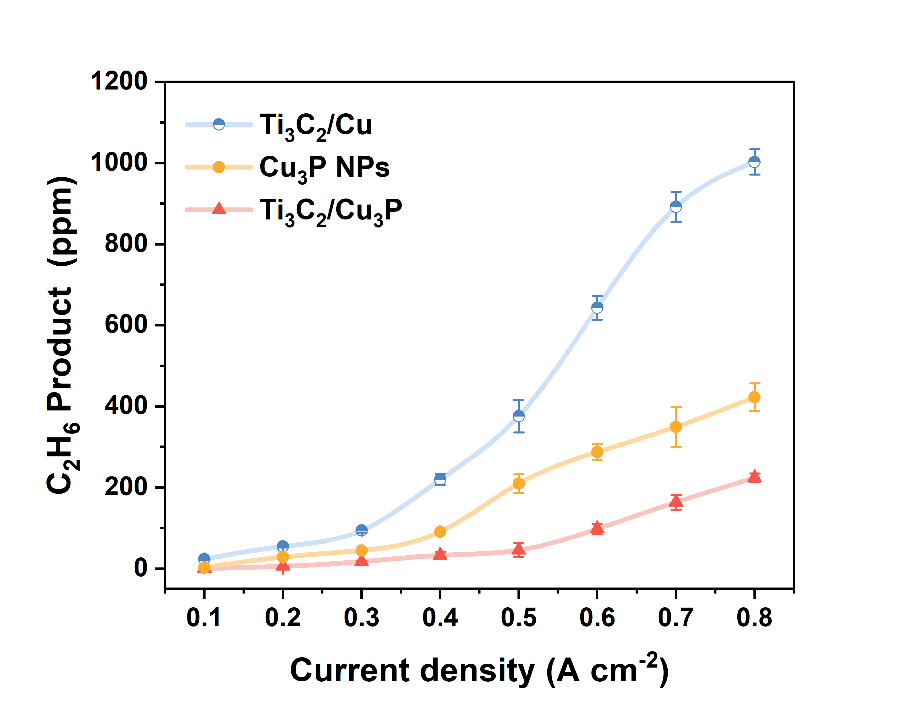


**Figure S17.** The content of C_2_H_6_ at the outlet over Ti_3_C_2_/Cu_3_P, Cu_3_P NPs, and Ti_3_C_2_/Cu at different current densities.

**Supplementary Note 6**. As current density was increased, the production of C_2_H_6_ also increased, which can be attributed to the excess H^*^ that could not be promptly consumed. It is important to note that the C_2_H_4_/C_2_H_6_ separation by cryogenic distillation is highly energy-intensive^[12–15]^.


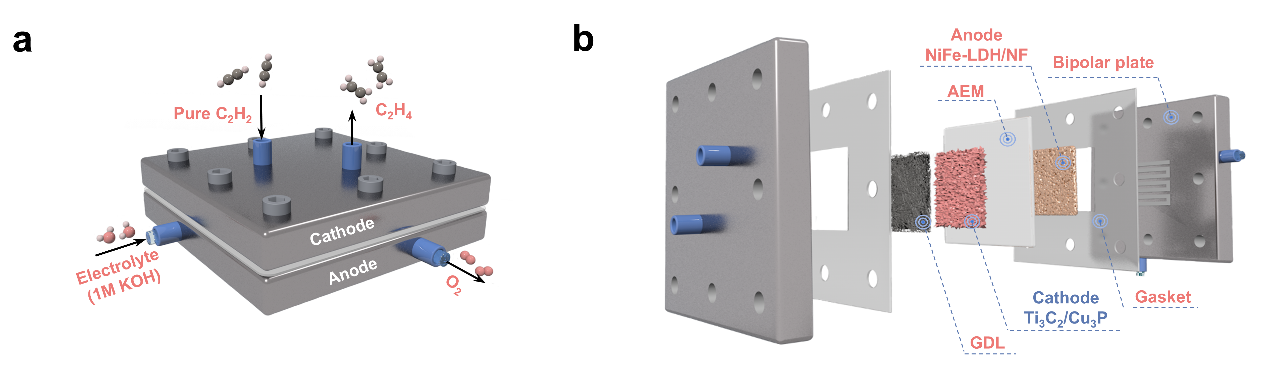


**Figure S18.** Schematic illustration of the EHAE process in a MEA.


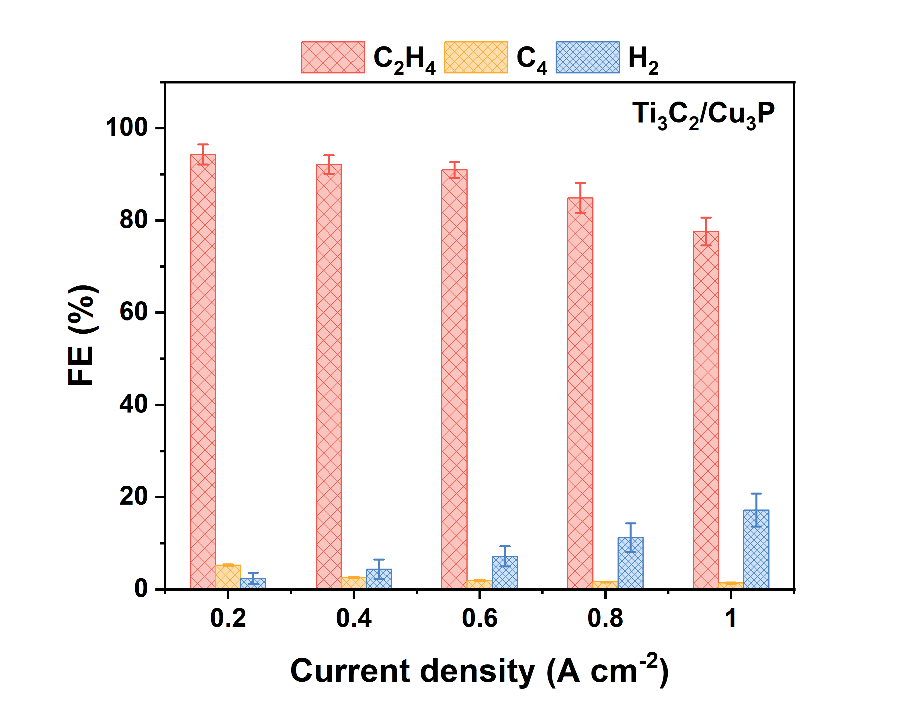


**Figure S19.** The FE of EHAE products at different current densities of Ti_3_C_2_/Cu_3_P in a 1cm^2^ MEA.


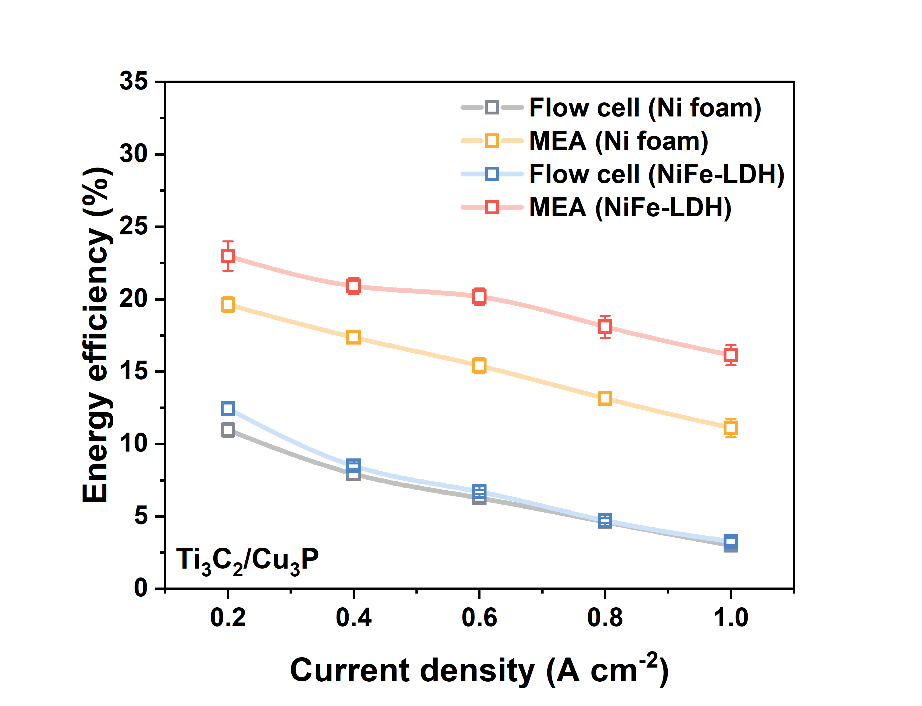


**Figure S20.** Comparison of energy efficiency with current density in different electrolyzers and anode for Ti_3_C_2_/Cu_3_P.


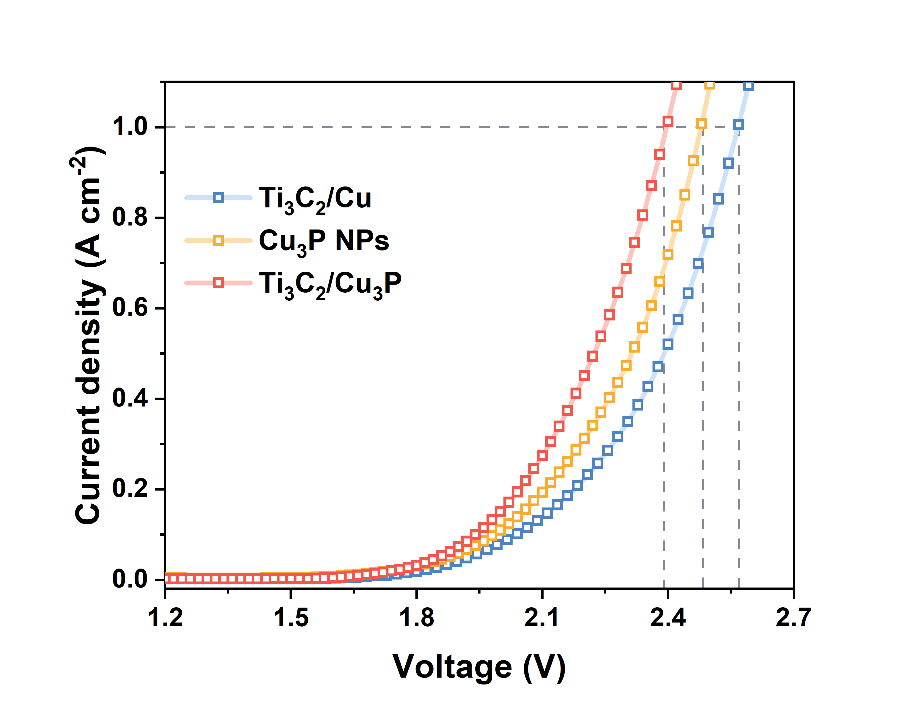


**Figure S21.** The LSV curves of Ti_3_C_2_/Cu_3_P, Cu_3_P NPs, and Ti_3_C_2_/Cu in a 1 cm^2^ MEA.

**Supplementary Note 7**. As shown in Figure S21, the cell voltage required for Ti_3_C_2_/Cu_3_P at 1 A cm^-2^ was only 2.39 V, lower than that of Cu_3_P NPs (2.48 V) and Ti_3_C_2_/Cu (2.57 V), indicating the superior ability of Ti_3_C_2_/Cu_3_P to drive C_2_H_2_ electroreduction.


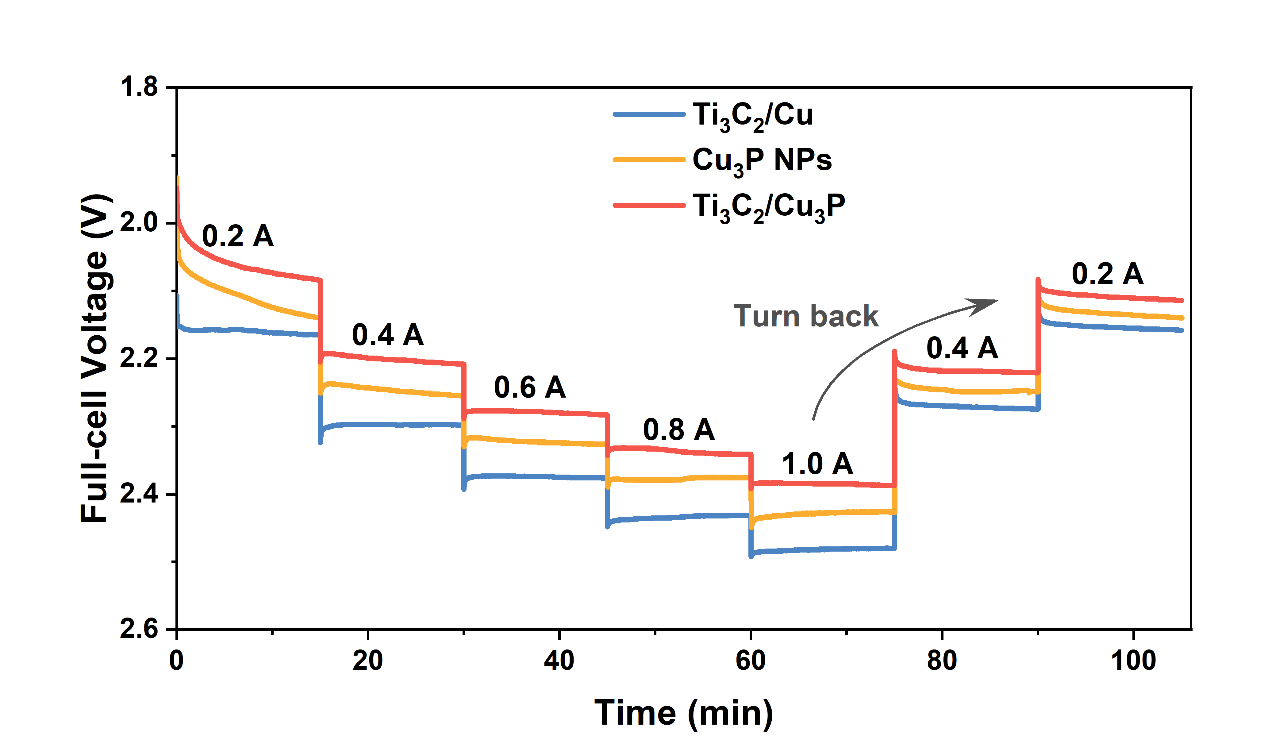


**Figure S22.** Changes of the full cell voltage at various applied currents.

**Supplementary Note 8**. Over a wide range of current densities (0.2-1.0 A cm^-2^), Ti_3_C_2_/Cu_3_P demonstrated reduced full-cell voltages compared to Cu_3_P NPs and Ti_3_C_2_/Cu, confirming its superior EHAE activity.


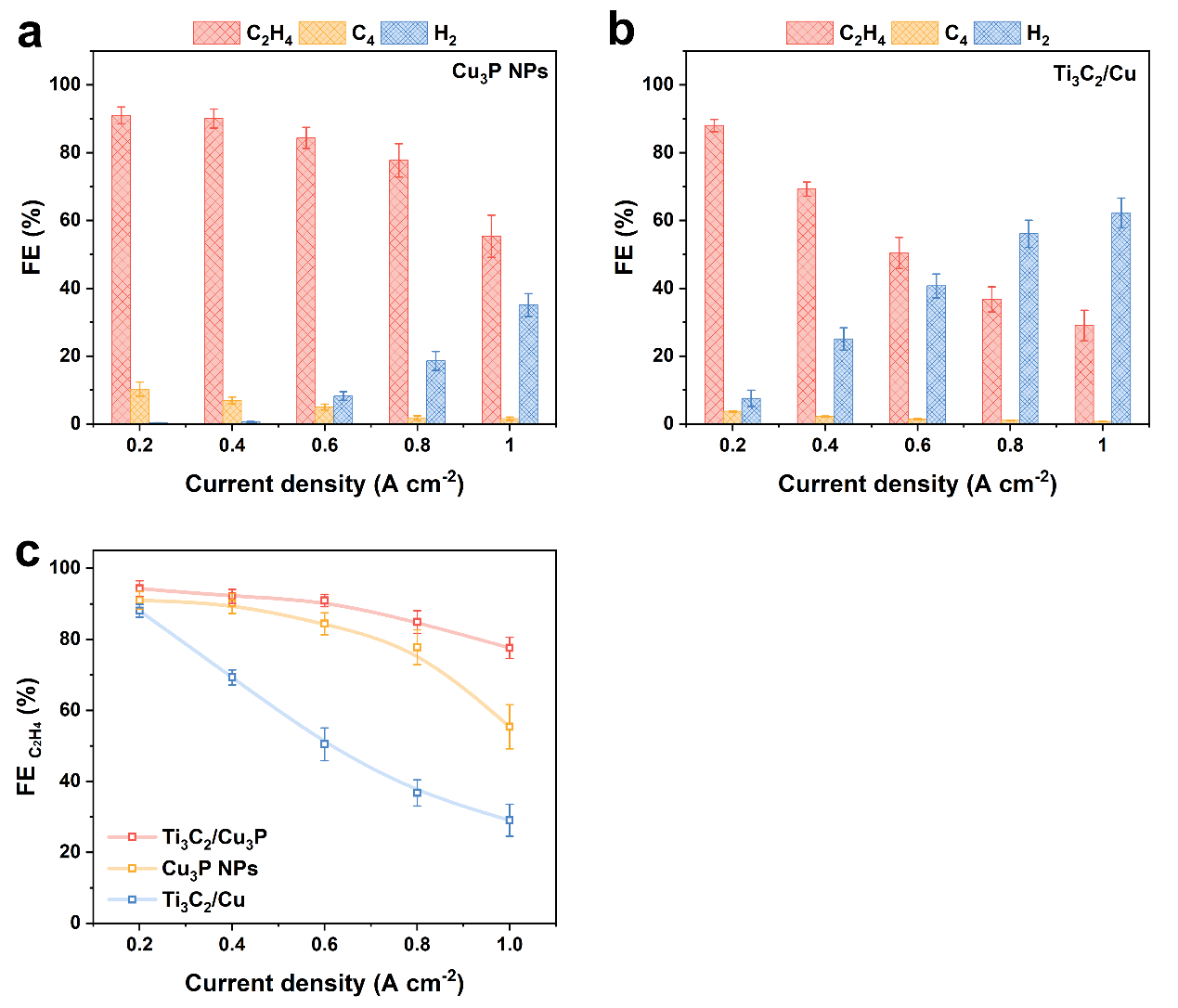
**Figure S23.** FE of EHAE products at different current densities for (**a**) Cu_3_P NPs and (**b**) Ti_3_C_2_/Cu. (**c**) Comparison of C_2_H_4_ FE at different current densities.


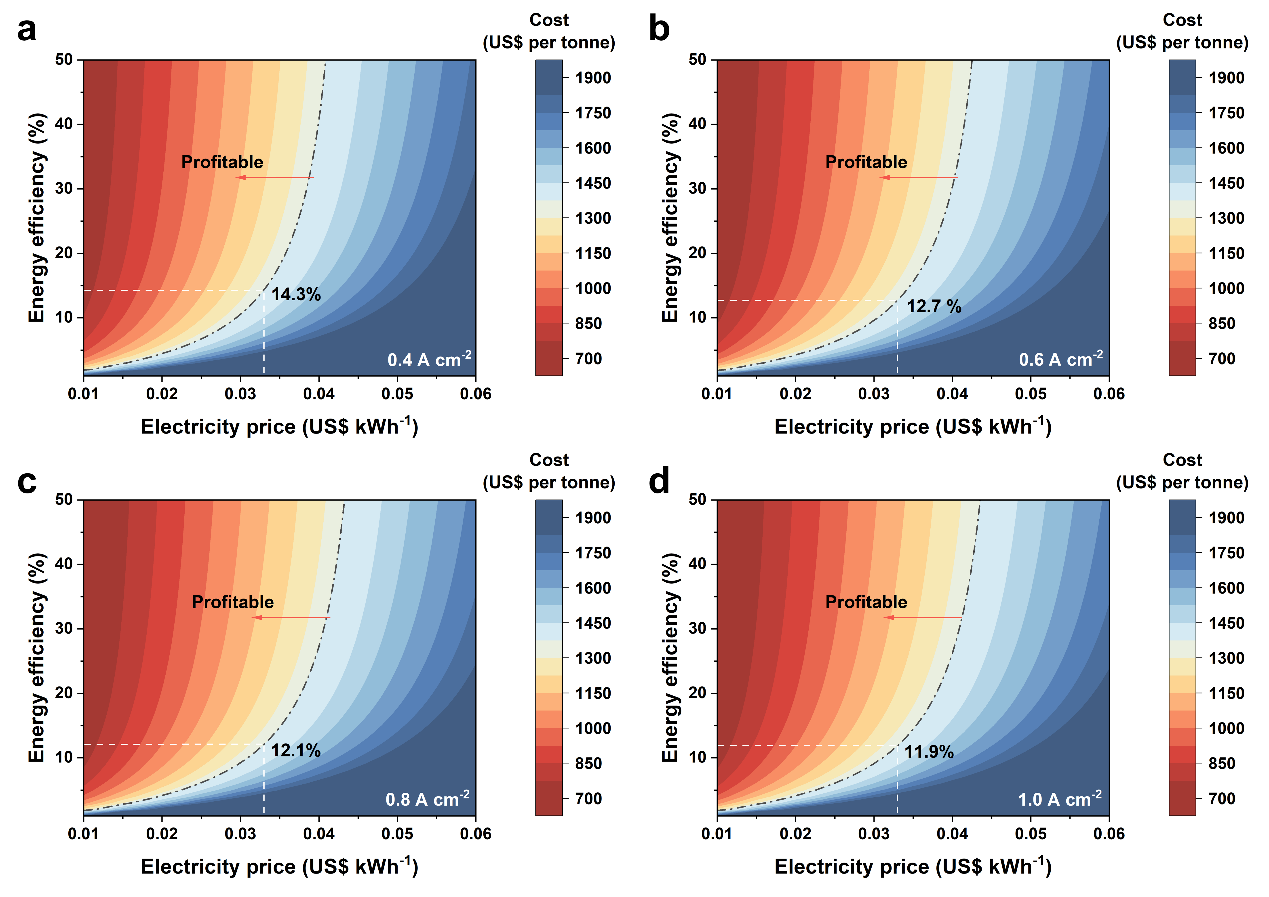


**Figure S24**. TEA results under different current densities from 0.4 to 1.0 A cm^-2^. (**a)** 0.4 A cm^-2^, (**b)** 0.6 A cm^-2^, (**c)** 0.8 A cm^-2^, and (**d)** 1.0 A cm^-2^.

**Supplementary Note 9**. The projected cost of the proposed EHAE process as a function of energy efficiency and electricity cost at different current densities. The area above the dashed-dotted grey line indicates a profitable region. The intersection of the white dashed lines in the figure represents the minimum energy efficiency required to achieve C_2_H_4_ profitability when the renewable electricity price is US$0.033 per kWh.


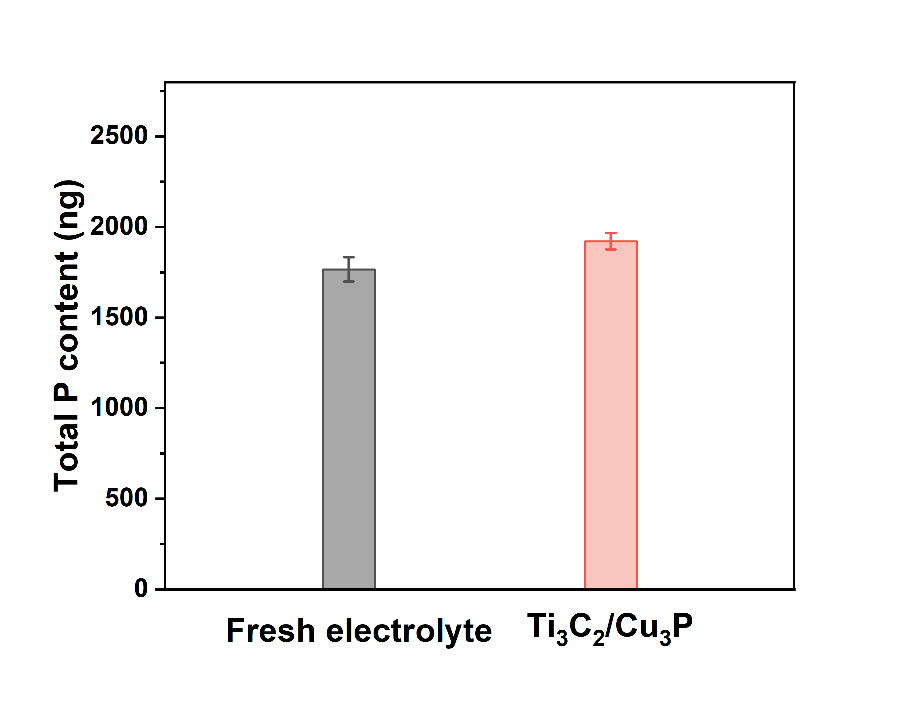


**Figure S25**. The dissolution of P in Ti_3_C_2_/Cu_3_P electrode at 1.0 A for 10 h. Note that the fresh electrolyte contains a small amount of P impurities.


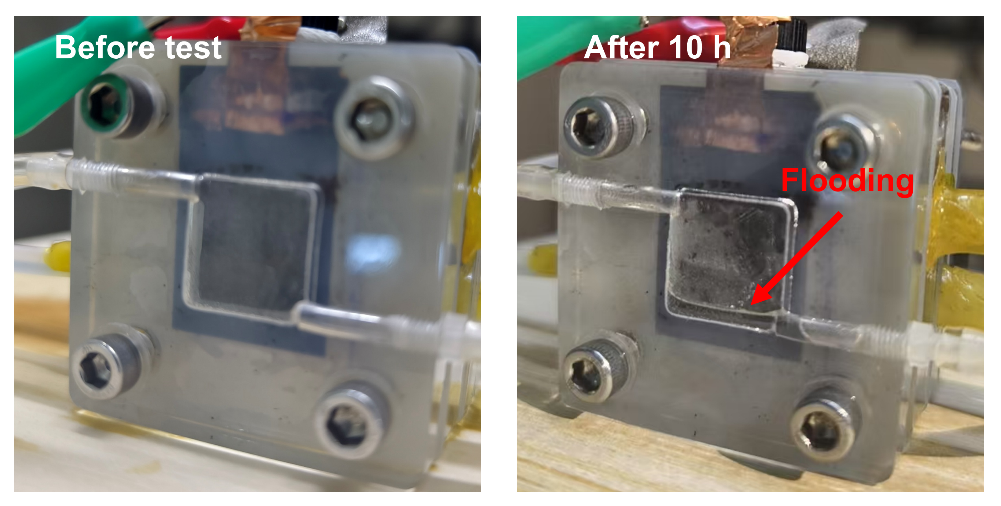


**Figure S26.** The digital image of the 4 cm^2^ flow cell before and after following the stability test.


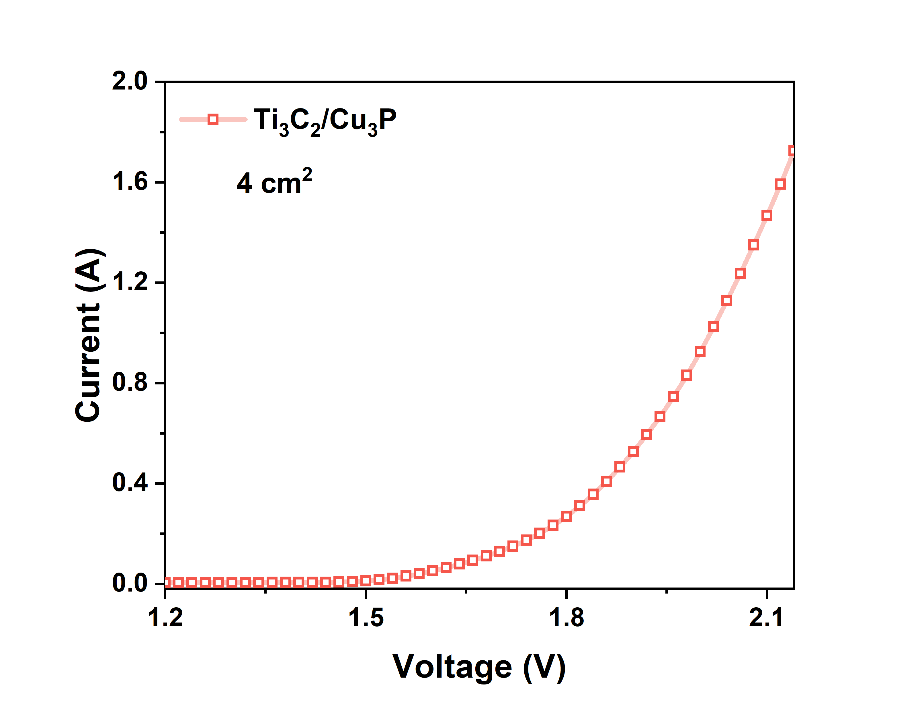


**Figure S27.** The LSV curve of Ti_3_C_2_/Cu_3_P in a 4 cm^2^ MEA.


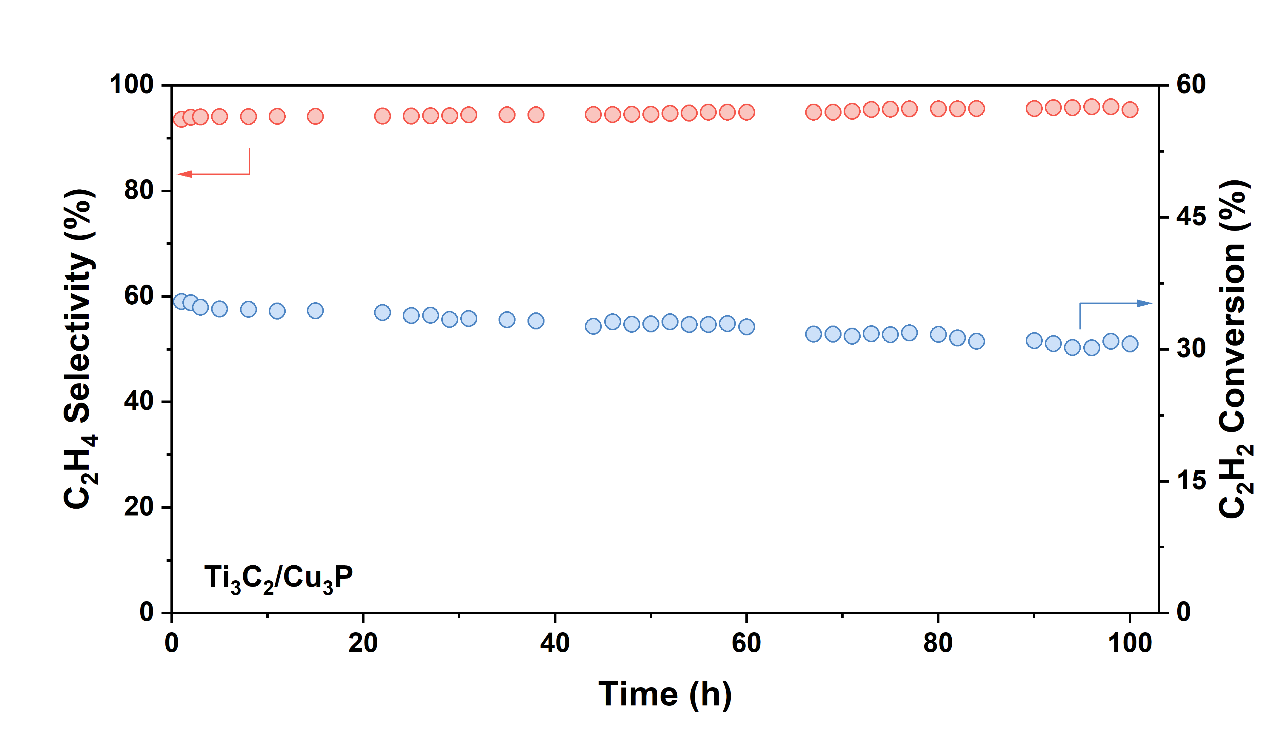


**Figure S28.** Time dependent C_2_H_2_ conversion and C_2_H_4_ Selectivity during stability test over Ti_3_C_2_/Cu_3_P.


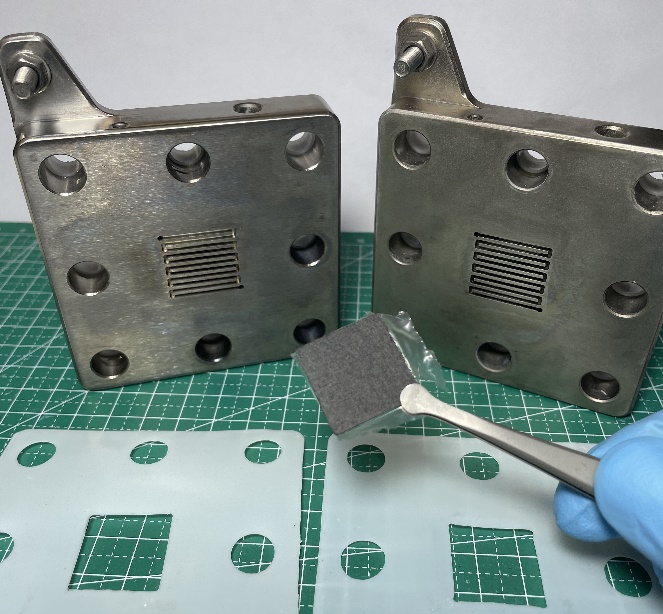


**Figure S29.** The digital image of 4 cm^2^ MEA after stability test.


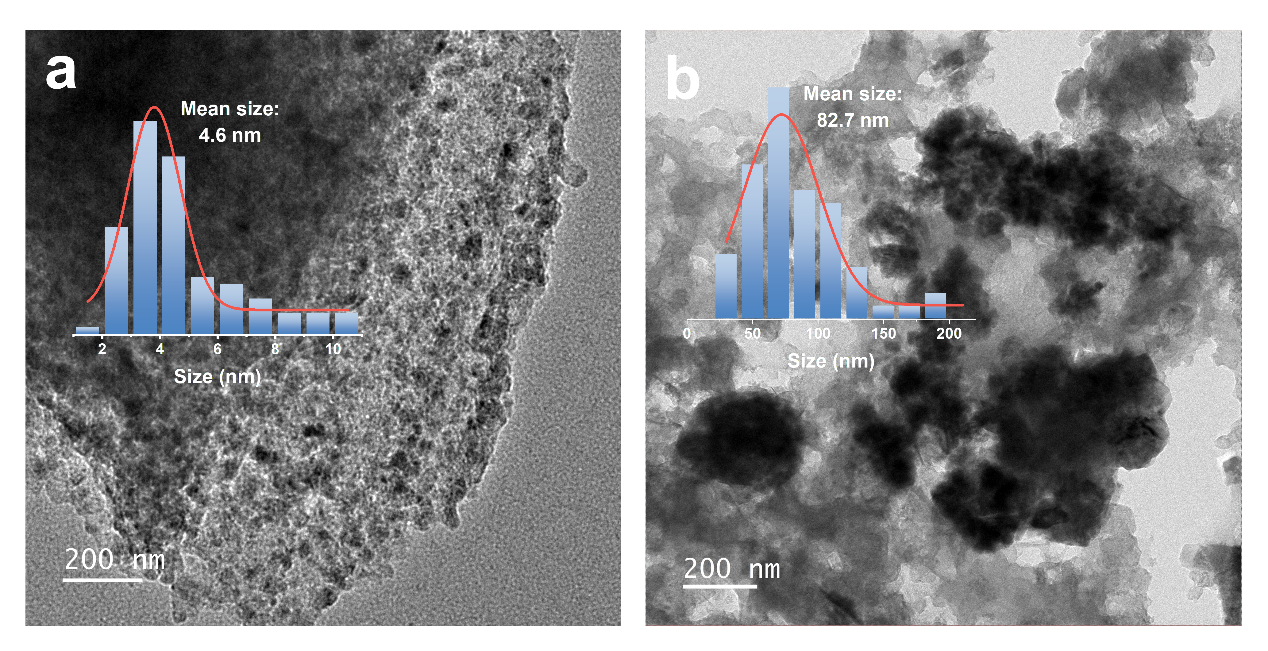


**Figure S30.** TEM images of Ti_3_C_2_/Cu (**a**) before and (**b**) after the long-term stability test.


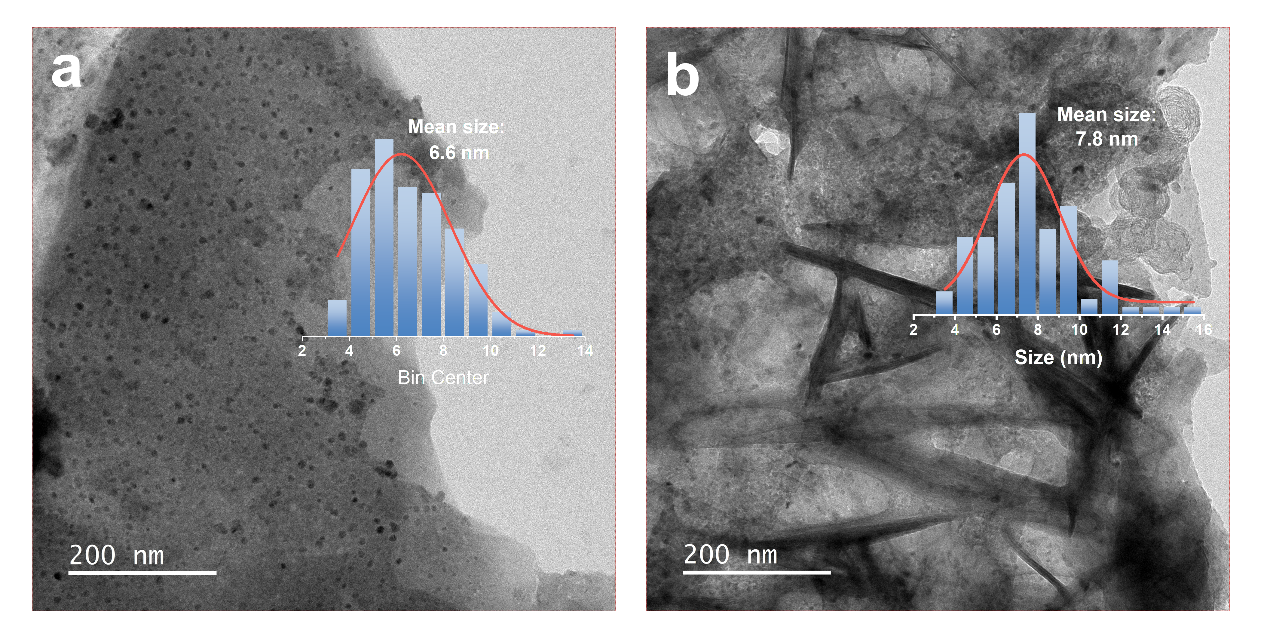


**Figure S31.** TEM images of Ti_3_C_2_/Cu_3_P (**a**) before and (**b**) after the long-term stability test.


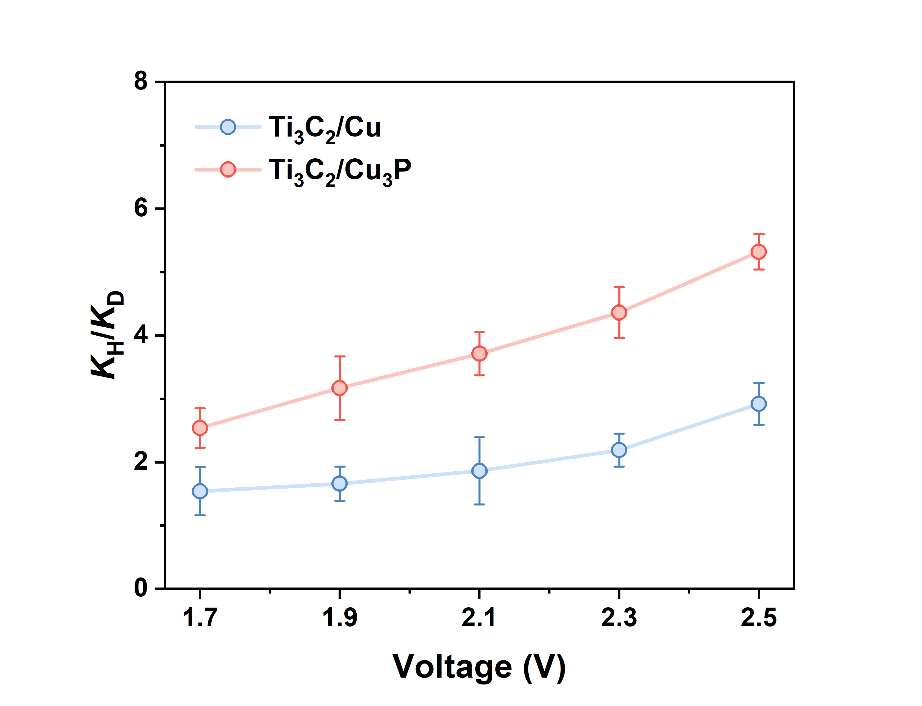


**Figure S32.** The KIE values of Ti_3_C_2_/Cu and Ti_3_C_2_/Cu_3_P at various applied potentials. Each data point represents the mean of three independent measurements, and the error bars indicate the corresponding standard deviations.


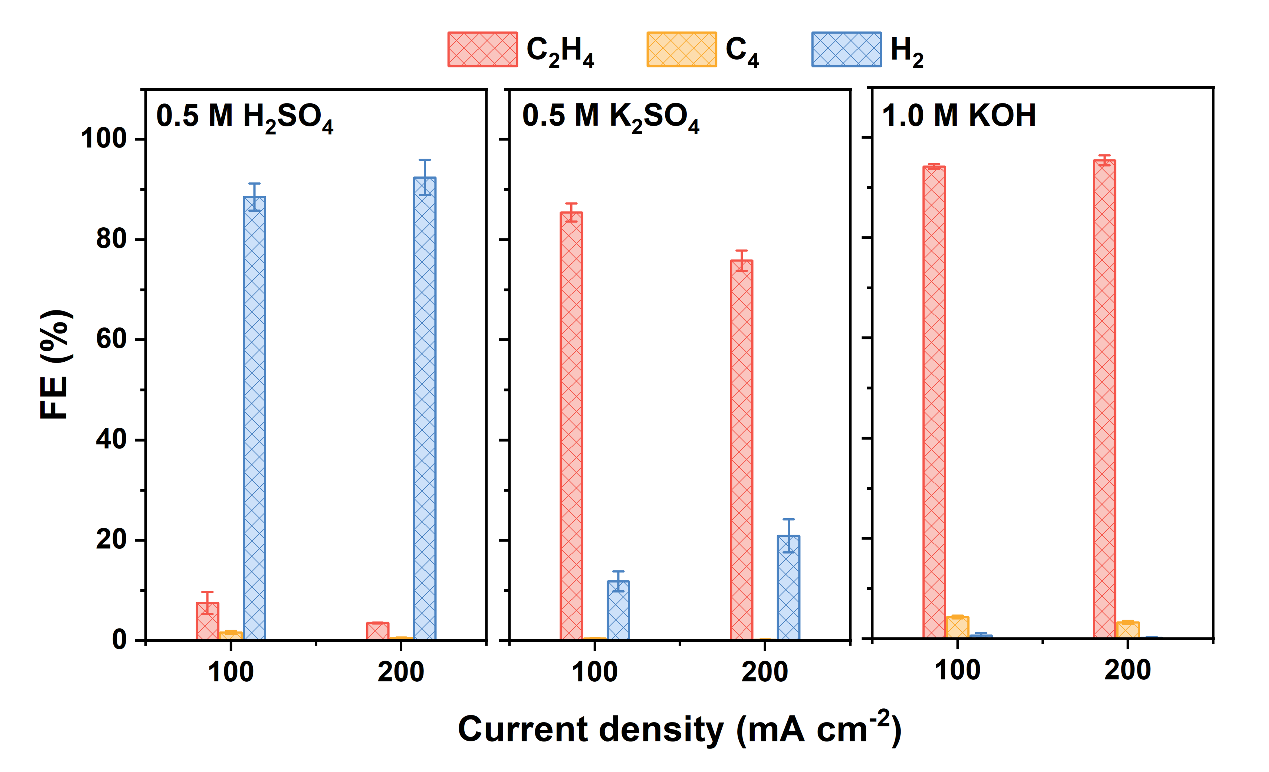


**Figure S33.** The EHAE performance of Ti_3_C_2_/Cu_3_P in different electrolytes.


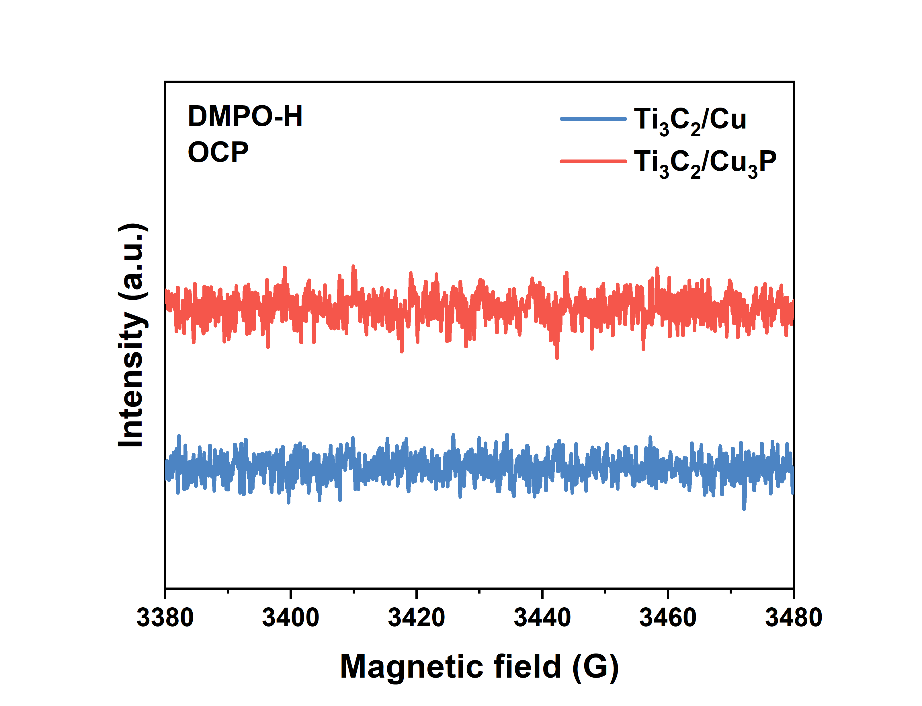


**Figure S34.** Quasi-*in-situ* EPR trapping of hydrogen radicals over Ti_3_C_2_/Cu and Ti_3_C_2_/Cu_3_P at open circuit potential (OCP).


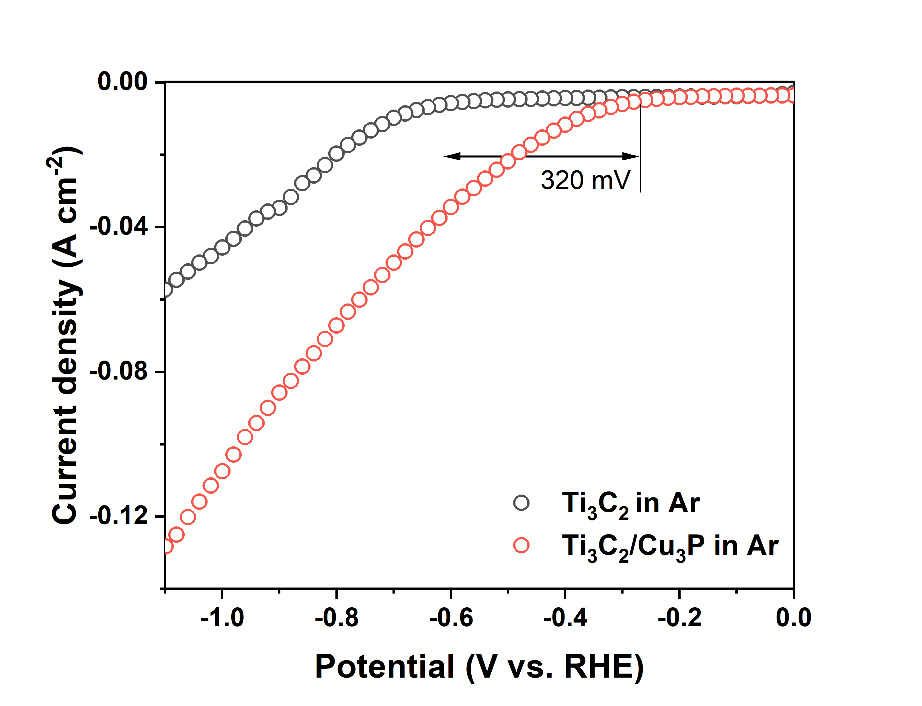


**Figure S35.** LSV curves for Ti_3_C_2_ and Ti_3_C_2_/Cu_3_P in Ar.

**Supplementary Note 10:** Ti_3_C_2_ delivers a more negative onset potential than Ti_3_C_2_/Cu_3_P, suggesting that water activation and H^*^ production occur preferentially on Cu_3_P particles rather than on the Ti_3_C_2_ support.


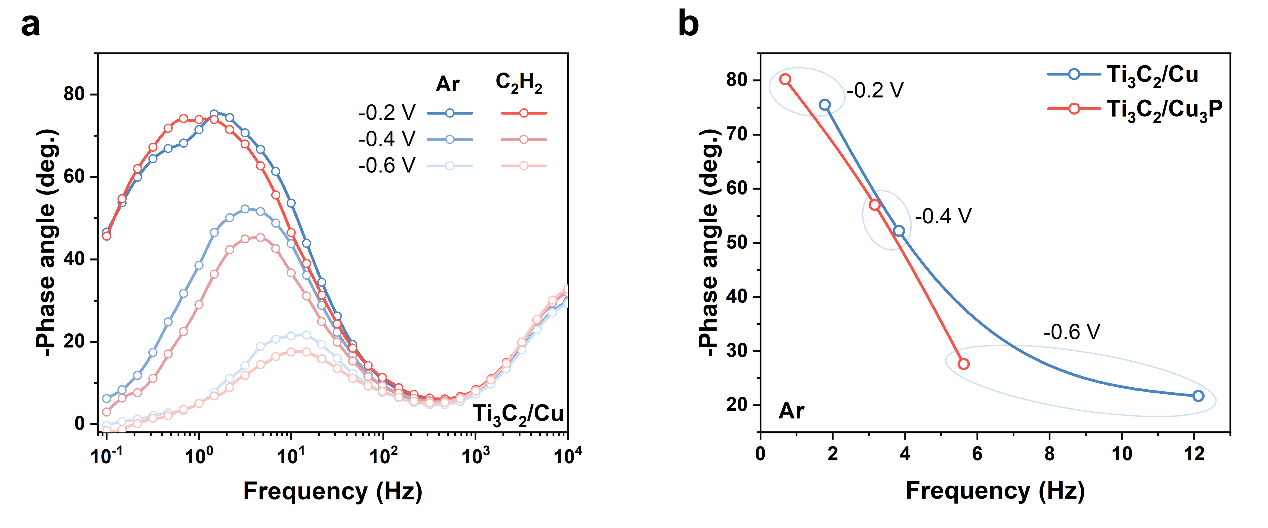


**Figure S36.** (a) Bode phase plots with Ar and C_2_H_2_ at various potentials for Ti_3_C_2_/Cu. (b) Potential-dependent Bode phase angle and frequency changes of Ti_3_C_2_/Cu and Ti_3_C_2_/Cu_3_P under Ar flow.


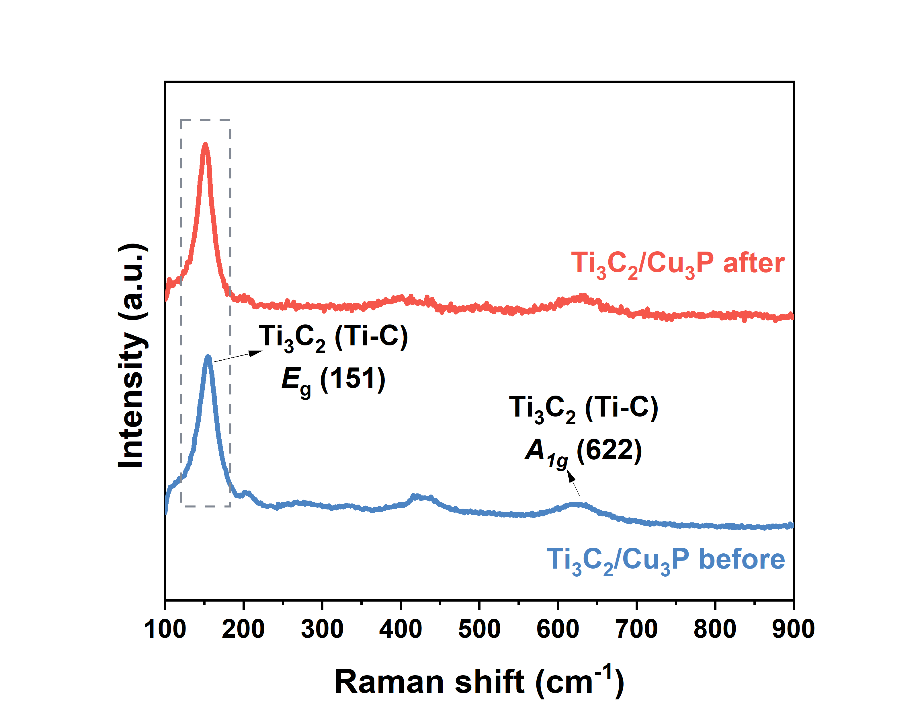


**Figure S37.** Raman spectra of Ti_3_C_2_/Cu_3_P electrode before and after reaction.


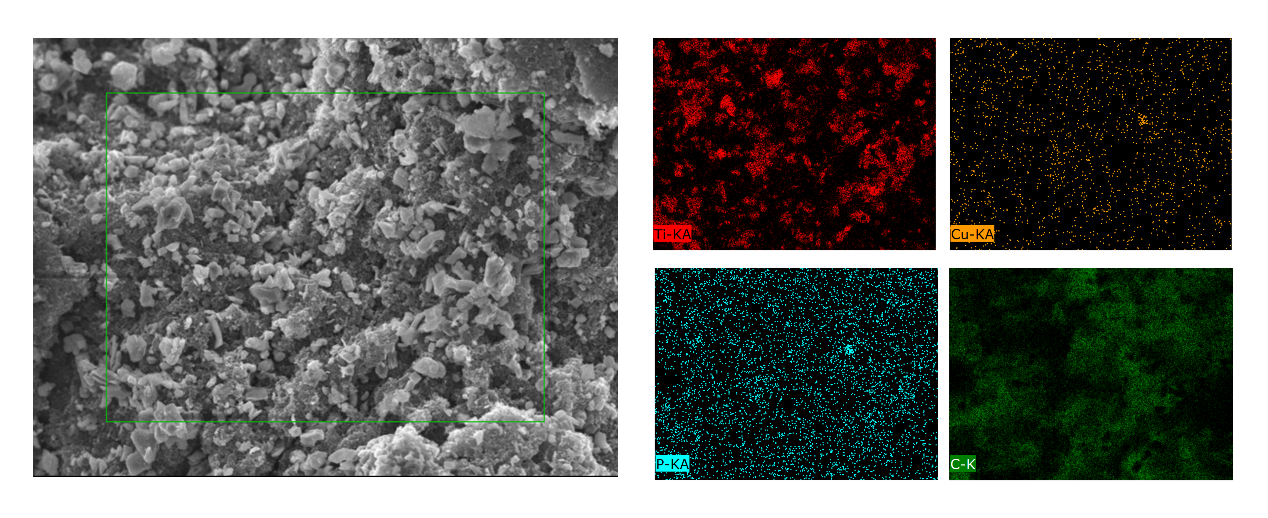


**Figure S38.** SEM image and corresponding EDS element mapping of Ti_3_C_2_/Cu_3_P electrode after the reaction.


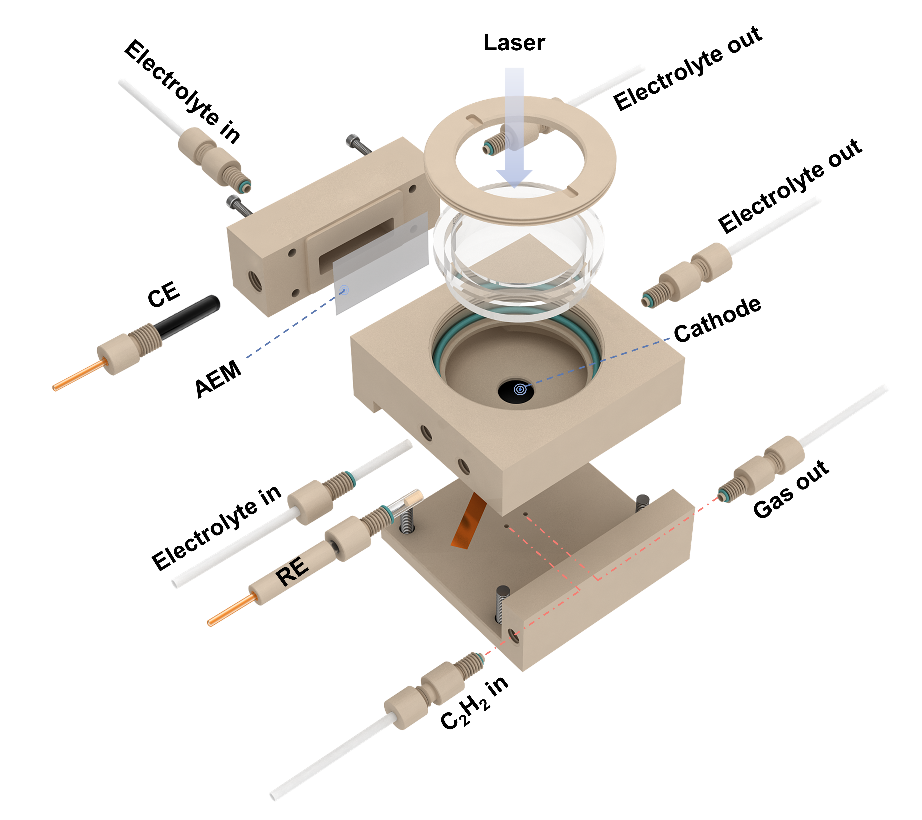


**Figure S39.** Schematic illustration of the in-situ Raman flow cell


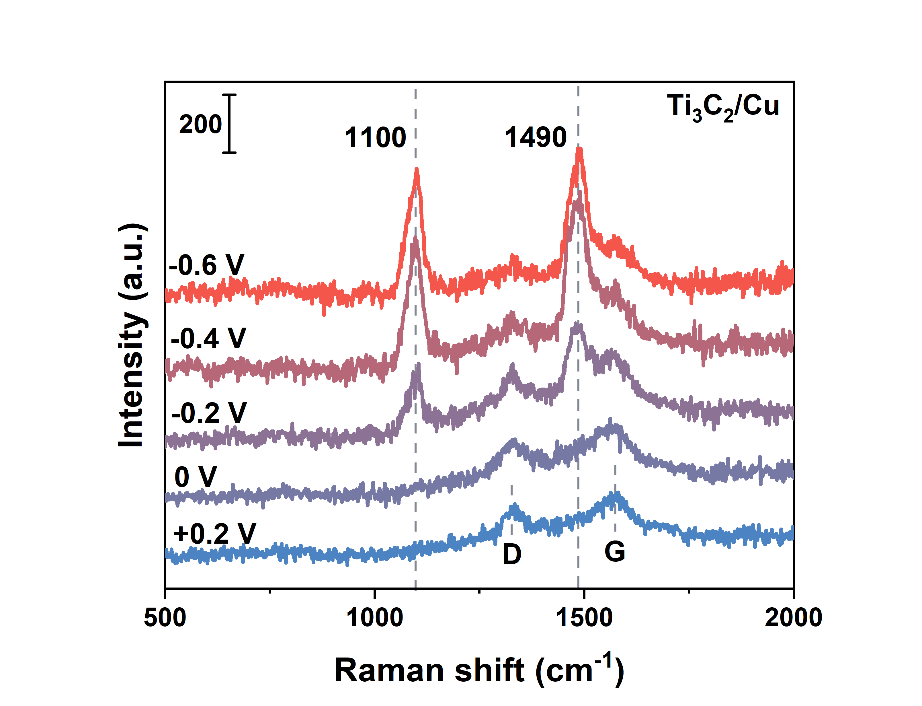


**Figure S40**. *In-situ* Raman spectra at different potentials on Ti_3_C_2_/Cu electrodes.


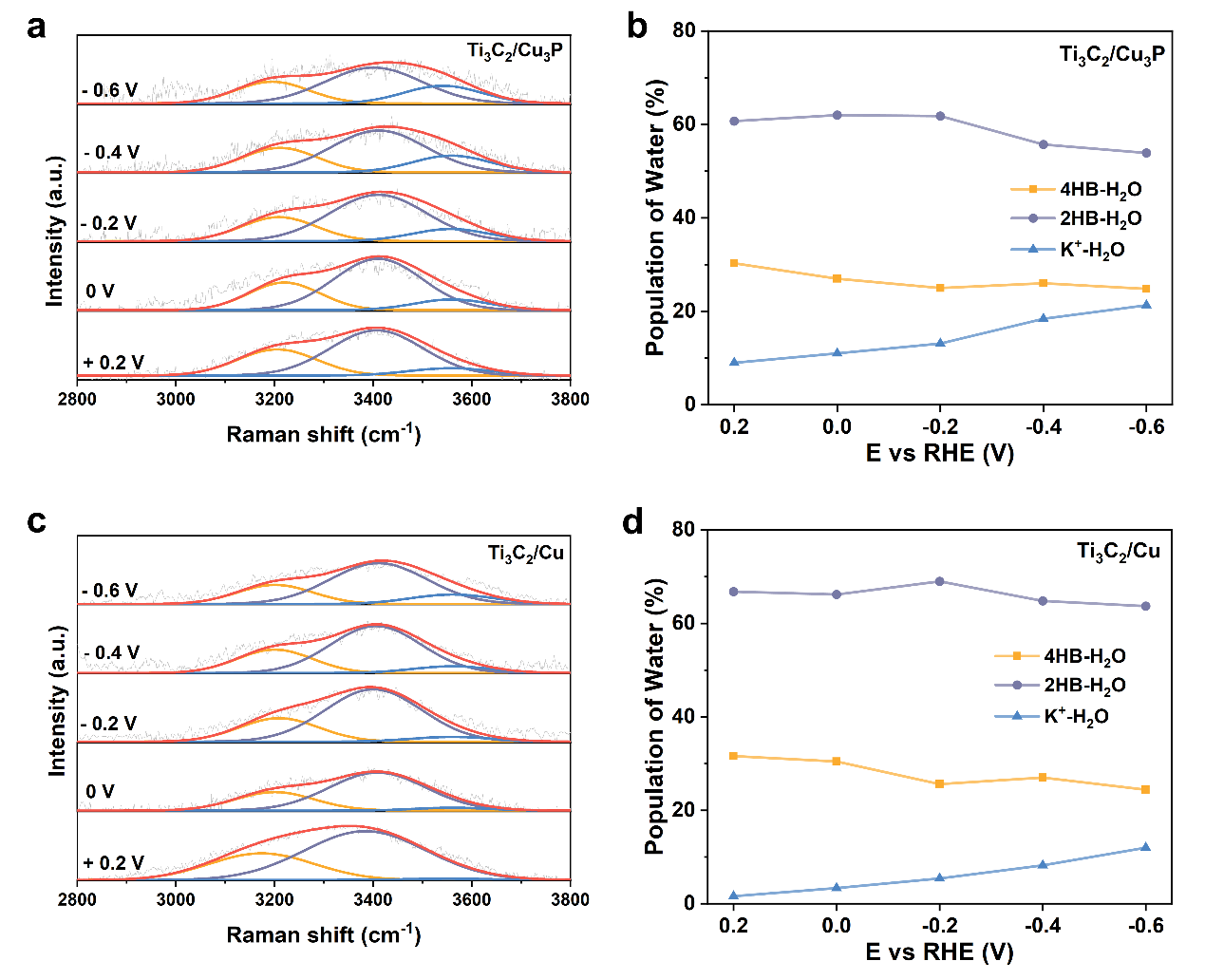


**Figure S41.** *In-situ* Raman spectra of interfacial water and Gaussian deconvolution of the O-H stretching region highlights three distinct water species on (a, b) Ti_3_C_2_/Cu_3_P and (c, d) electrodes.


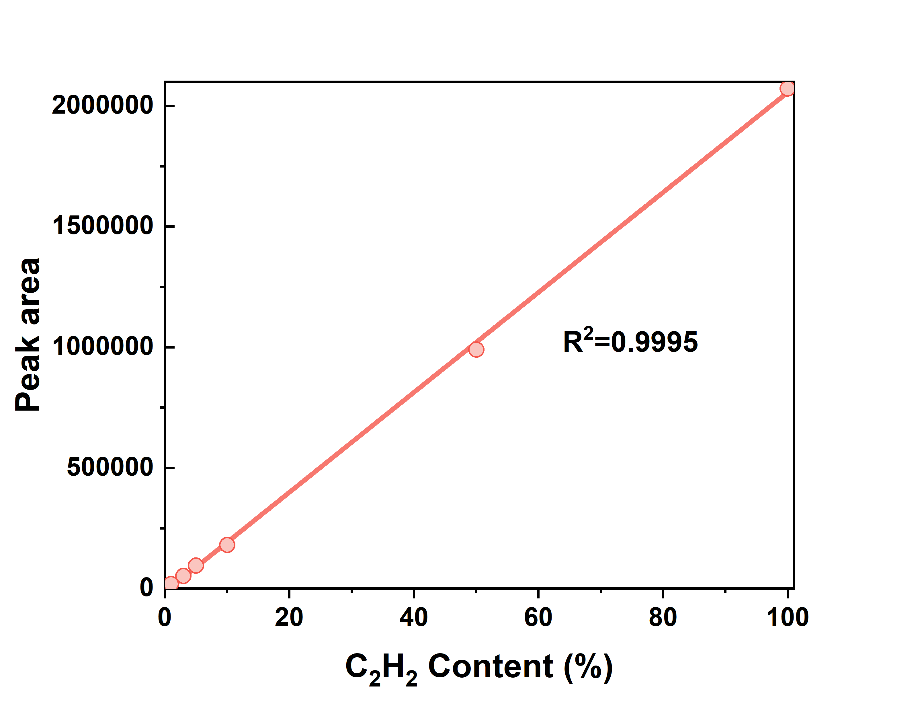


**Figure S42.** The standard curve of C_2_H_2_ with different volume fractions.

Tables S1-S7

**Table S1*.*** Comparison of EHAE performance under pure C_2_H_2_ flow in three-electrode flow cell.

| Catalysts | Electrolyte | Cathode current density (A cm^-2^) | FE of C_2_H_4_ (%) | Reference |
| --- | --- | --- | --- | --- |
| Ti_3_C_2_/Cu_3_P | **1.0 M KOH** | **0.1** | **93.5** | **This work** |
|  |  | **0.2** | **95.3** |  |
|  |  | **0.3** | **95.9** |  |
|  |  | **0.4** | **96.3** |  |
|  |  | **0.5** | **97.1** |  |
|  |  | **0.6** | **94.8** |  |
|  |  | **0.7** | **94.2** |  |
|  |  | **0.8** | **84.4** |  |
| Cu NDs | 1.0 M KOH | 0.5 | 90.4 | *Nat. Commun.***14**, 2137 **(2023)** |
| ED-16 | 1.0 M KOH | 0.4 | 95.1 | *Angew. Chem. Int. Ed.* **63**, e202405943 (**2024**) |
| ED-Cu NPs | 1.0 M KOH | 0.5 | 97.7 | *Nat. Sustain.* **6**,  827-837 **(2023)** |
| Ag NWs | 1.0 M KOH | 0.219 | 99 | *CCS Chem.***5**, 200-208 **(2022)** |

**Table S2*.*** Comparison of EHAE performance in MEA and flow cell systems.

| System | Anolyte | Full-cell voltage (V) | Current density (A cm^-2^) | FE of C_2_H_4_ (%) | Energy efficiency (%) |
| --- | --- | --- | --- | --- | --- |
| MEA | 1.0 M KOH | 2.04 | 0.2 | 94.3 | 23.0 |
|  |  | 2.19 | 0.4 | 92.1 | 20.9 |
|  |  | 2.24 | 0.6 | 90.9 | 20.2 |
|  |  | 2.33 | 0.8 | 84.8 | 18.1 |
|  |  | 2.39 | 1.0 | 77.6 | 16.1 |
| Flow cell | 1.0 M KOH | 3.81 | 0.2 | 95.3 | 12.4 |
|  |  | 5.64 | 0.4 | 96.3 | 8.5 |
|  |  | 7.03 | 0.6 | 94.8 | 6.7 |
|  |  | 8.89 | 0.8 | 84.4 | 4.7 |
|  |  | 10.85 | 1.0 | 71.3 | 3.3 |

**Table S3*.*** Comparison of the reported full-cell energy efficiency.

| Catalysts | Cathode current density (A cm^-2^) | Energy efficiency (%) | Reference |
| --- | --- | --- | --- |
| Ti_3_C_2_/Cu_3_P | **0.2** | **23.0** | **This work** |
|  | **0.4** | **20.9** |  |
|  | **0.6** | **20.2** |  |
|  | **0.8** | **18.1** |  |
|  | **1.0** | **16.1** |  |
| V-Cu NCs | 0.3 | 9.6 | *CCS Chem.* Just Published (**2024**) |
|  | 0.5 | 7.2 |  |
|  | 0.7 | 6.2 |  |
|  | 0.9 | 5.0 |  |
| CuO_1-x_ NRs | 0.2 | 12.4 | *Adv. Mater.*, **36**, 2408681 (**2024**) |
|  | 0.4 | 8.4 |  |
|  | 0.6 | 6.6 |  |
|  | 0.8 | 5.5 |  |
|  | 1.0 | 4.6 |  |
| 2TIm | 0.15 | ~14.6 | *Nat. Chem.***16**, 893-900 **(2024)** |
| ED-2 | 0.2 | ~14.8 | *Angew. Chem. Int. Ed.* **63**, e202405943 (**2024**) |
|  | 0.4 | ~12.1 |  |
|  | 0.6 | ~9.7 |  |
|  | 0.8 | ~7.5 |  |
|  | 1.0 | ~6.2 |  |
| ED-Cu NPs | 0.2 | ~19.7 | *Nat. Sustain.* **6**,  827-837 **(2023)** |
|  | 0.3 | ~16.5 |  |
|  | 0.4 | ~14.4 |  |
|  | 0.5 | ~11.0 |  |
| Ag NWs | 0.09 | ~15.1 | *CCS Chem.***5**, 200-208 **(2022)** |

**Table S4*.*** Production cost calculation of the EHAE process.

| C_2_H_2_ Costs | |
| --- | --- |
| Capital cost ($ per ton C_2_H_2_) | 125.4 |
| Coal cost ($ per ton C_2_H_2_) | 128.6 |
| Hydrogen cost ($ per ton C_2_H_2_) | 153.8 |
| Electricity cost ($ per ton C_2_H_2_) | 462.0 |
| Separation cost ($ per ton C_2_H_2_) | 138.6 |
| Cost of C_2_H_2_ from coal arc plasma ($ per ton C_2_H_2_) | **1,008.4** |
|  | |
| Electrolyzer Cost ($ m^-2^) | 3,462.1 |
| Total Current (A) | 8,765,498.7 |
| Needed Electrolyzer Area (m^2^) | 1,460.9 |
| Required Daily Power (MW day^-1^) | 19.6 |
| C_2_H_2_ Inlet Flow Rate (kg day^-1^) | 485,908.6 |
| C_2_H_2_ Outlet Flow Rate (m^3^ h^-1^) | 13,670.5 |
| C_2_H_4_ Outlet Flow Rate (m^3^ h^-1^) | 3,333.3 |
| H_2_ Outlet Flow Rate (m^3^ h^-1^) | 220.5 |
| C_4_ Outlet Flow Rate (m^3^ h^-1^) | 0.28 |
| Electrochemical Capital Cost | |
| Electrolyzer ($ per ton C_2_H_4_) | 7.0 |
| Balance of Plant ($ per ton C_2_H_4_) | 3.8 |
| Separation Equipment ($ per ton C_2_H_4_) | 20.3 |
| Electrochemical Operating Cost | |
| Electricity ($ per ton C_2_H_4_) | 155.5 |
| Maintenance ($ per ton C_2_H_4_) | 3.5 |
| Separation ($ per ton C_2_H_4_) | 34.2 |
| Electrochemical Process Material Cost | |
| Water ($ per ton C_2_H_4_) | 0.5 |
| Catalyst ($ per ton C_2_H_4_) | 0.5 |
| Membrane ($ per ton C_2_H_4_) | 15.4 |
| C_2_H_2_ ($ per ton C_2_H_4_) | 1008.4 |
| Total Cost of Production ($ ton^-1^) | **1249.1** |

**Table S5*.*** The subdivided cost analysis of the EHAE process.

| Total Costs | Specific Items | Costs ($ ton^-1^) | Percentage (%) |
| --- | --- | --- | --- |
| Operating Costs  (63.6%) | **Electricity** | 617.5 | **49.5** |
|  | **Separation** | 172.8 | **13.8** |
|  | **Maintenance** | 3.5 | **0.28** |
| Material Costs  (23.9%) | **Hydrogen** | 153.8 | **12.3** |
|  | **Coal** | 128.6 | **10.3** |
|  | **Water** | 0.5 | **0.04** |
|  | **Membrane** | 15.4 | **1.24** |
|  | **Catalyst** | 0.5 | **0.04** |
| Capital Costs  (12.5%) | **Electrolyzer** | 7.0 | **0.6** |
|  | **Balance of Plant** | 3.8 | **0.3** |
|  | **Reactor** | 145.7 | **11.7** |

**Table S6*.*** Calculated Gibbs free energies of water dissociation for Ti_3_C_2_/Cu and Ti_3_C_2_/Cu_3_P.

| System | Ti_3_C_2_/Cu  Free energy (eV) | Ti_3_C_2_/Cu_3_P  Free energy (eV) |
| --- | --- | --- |
| ^*^H_2_O | -0.52 | -0.61 |
| TS | 0.27 | -0.11 |
| ^*^H+^*^OH | -0.23 | -0.45 |

**Table S7*.*** Calculated Gibbs free energies of EHAE for Ti_3_C_2_/Cu and Ti_3_C_2_/Cu_3_P.

| System | Ti_3_C_2_/Cu  Free energy (eV) | Ti_3_C_2_/Cu_3_P  Free energy (eV) |
| --- | --- | --- |
| C_2_H_2_^*^ | -0.97 | -1.54 |
| C_2_H_2_^*^+H^*^ | -0.90 | -1.62 |
| C_2_H_3_^*^ | -1.19 | -1.26 |
| C_2_H_3_^*^+H^*^ | -1.12 | -1.34 |
| C_2_H_4_^*^ | -1.38 | -1.96 |
| C_2_H_4_^*^+H^*^ | -1.31 | -2.03 |
| C_2_H_5_^*^ | -1.95 | -1.61 |
| C_2_H_5_^*^+H^*^ | -1.88 | -1.69 |
| C_2_H_6_ (g) | -2.62 | -2.62 |

References

[1] W. J. Teh, E. Romeo, S. Xi, B. Rowley, F. Illas, F. Calle-Vallejo, B. S. Yeo, *Nat. Catal.* **2024**, *7*, 1382–1393.

[2] R. Shi, Z. Wang, Y. Zhao, G. I. N. Waterhouse, Z. Li, B. Zhang, Z. Sun, C. Xia, H. Wang, T. Zhang, *Nat. Catal.* **2021**, *4*, 565–574.

[3] B.-H. Zhao, F. Chen, M. Wang, C. Cheng, Y. Wu, C. Liu, Y. Yu, B. Zhang, *Nat. Sustain.* **2023**, *6*, 827–837.

[4] IRENA, *Renewable Generation Costs in 2023*, **2024**.

[5] L. Huang, D. Bao, Y. Jiang, Y. Zheng, S. Qiao, *Angew. Chemie Int. Ed.* **2024**, *63*, e202405943.

[6] H. J. Jiang, T. C. Underwood, J. G. Bell, S. Ranjan, D. Sasselov, G. M. Whitesides, *Proc. Natl. Acad. Sci.* **225AD**, *122*, e2505151122.

[7] H. Shin, K. U. Hansen, F. Jiao, *Nat. Sustain.* **2021**, *4*, 911–919.

[8] P. Thiruvenkataswamy, Safety and Techno-Economic Analysis of Ethylene Technologies, Texas A&M University, **2015**.

[9] Z. Wu, J. Zhang, Q. Guan, X. Liu, H. Xiong, S. Chen, W. Hong, D. Li, Y. Lei, S. Deng, J. Wang, G. Wang, *Adv. Mater.* **2024**, *36*, 2408681.

[10] Y. Li, H. Shao, Z. Lin, J. Lu, L. Liu, B. Duployer, P. O. Å. Persson, P. Eklund, L. Hultman, M. Li, K. Chen, X. H. Zha, S. Du, P. Rozier, Z. Chai, E. Raymundo-Piñero, P. L. Taberna, P. Simon, Q. Huang, *Nat. Mater.* **2020**, *19*, 894–899.

[11] Y. Bai, C. Liu, T. Chen, W. Li, S. Zheng, Y. Pi, Y. Luo, H. Pang, *Angew. Chemie-International Ed.* **2021**, *60*, 25318–25322.

[12] K. J. Chen, D. G. Madden, S. Mukherjee, T. Pham, K. A. Forrest, A. Kumar, B. Space, J. Kong, Q. Y. Zhang, M. J. Zaworotko, *Science.* **2019**, *366*, 241–246.

[13] L. Li, R. Lin, R. Krishna, H. Li, S. Xiang, H. Wu, J. Li, W. Zhou, B. Chen, *Science (80-. ).* **2018**, *362*, 443–446.

[14] M. Yang, F. You, *Ind. Eng. Chem. Res.* **2017**, *56*, 4038–4051.

[15] X. Zhang, H. He, Y. Gan, Y. Wang, N. Huang, P. Liao, J. Zhang, X. Chen, *Angew. Chemie Int. Ed.* **2024**, *63*, e202317648.
